# Time Series Segmentation in Adaptive Optics Ophthalmoscopy With Application to Flicker-Induced Vasodilation

**DOI:** 10.1167/tvst.15.10.8

**Published:** 2026-10-07

**Authors:** Alireza Vard, Abir Aissa, Florence Rossant, Isabelle Bloch, Daniela Castro-Farias, Hugues Chabriat, Michel Paques

**Affiliations:** 1 Medical Image and Signal Processing Research Center, School of Advanced Technologies in Medicine, Isfahan University of Medical Sciences, Isfahan, Iran; 2 Télécom Paris, LTCI, Institut Polytechnique de Paris, Palaiseau, France; 3 Isep, Institut Supérieur d'Electronique de Paris, Paris, France; 4 Sorbonne Université, CNRS, LIP6, Paris, France; 5 Clinical Investigation Center 1423, Quinze-Vingts Hospital, Sorbonne Université, INSERM, Paris, France; 6 Referral Centre CERVCO at the University Hospital Lariboisière in APHP (University Paris Cité), France

**Keywords:** segmentation, active contour models, deep learning, adaptive optics, micro vessel alterations, flicker images, vasomotion, neurovascular coupling

## Abstract

**Purpose:**

To develop an automated image processing pipeline to segment retinal arterial walls in a series of adaptive optics ophthalmoscopy (AOO) images, overcoming difficulties related to various quality levels within the series, to accurately evaluate morphometric differences.

**Methods:**

We first assess the quality of AOO images by deep learning in order to select the best quality image in the sequence. Then, we segment this image by combining deep learning and dedicated active contour models. Finally, we segment the other images of the sequence using the best image segmentation as a regularization exploiting the temporal continuity to compensate for lower quality.

**Results:**

We applied the proposed method to flicker series, a challenging use-case given the image quality heterogeneity. The quality assessment method achieves a Spearman correlation coefficient of 0.86, demonstrating very good agreement with the quality ranking established by the medical expert. The automatic segmentation method results in accurate segmentations with a mean root mean square error of 0.5 pixel and 1.8 pixels for the inner and outer borders of the arterial wall, respectively. This leads to accurate and unbiased estimates of the wall to lumen ratio.

**Conclusions:**

Our algorithm can be applied as a reliable and useful computer-aided tool to process large time series databases in AOO, and can help experts in longitudinal monitoring applications.

**Translational Relevance:**

For the first time, AOO image series can be processed with low supervision to accurately monitor morphometric changes in retinal vascularization.

## Introduction

Clinical imaging of the retina allows ophthalmologists to noninvasively document human micro-vessels. These vessels may be altered by multiple types of ocular or systemic disorders. The advent of adaptive optics ophthalmoscopy (AOO) enabled clinicians and researchers to observe and measure the morphometric parameters of retinal vessels at the micrometric scale.^1^ Such measures might be of particular interest to explore multiple conditions,^2^^–^^4^ including various cerebrovascular disorders such as cerebral autosomal dominant arteriopathy with subcortical infarcts and leukoencephalopathy (CADASIL), affecting specifically the cerebral and retinal microvasculature.^5^

The extraction of morphometric parameters relies on AOO image segmentation, to delineate the vessel lumen and the arterial walls, which is a very challenging task. Indeed, AOO images exhibit irregular local quality and inherent blur, partly because retinal vessels are not entirely planar. In addition, the thin vascular walls are very poorly contrasted against the highly textured background. Complex vessel configurations, such as arteriovenous crossings, introduce additional blur and distortions arising from depth variations. Collectively, these factors pose significant challenges for automatic vessel segmentation (Fig. 1).

**Figure 1.**
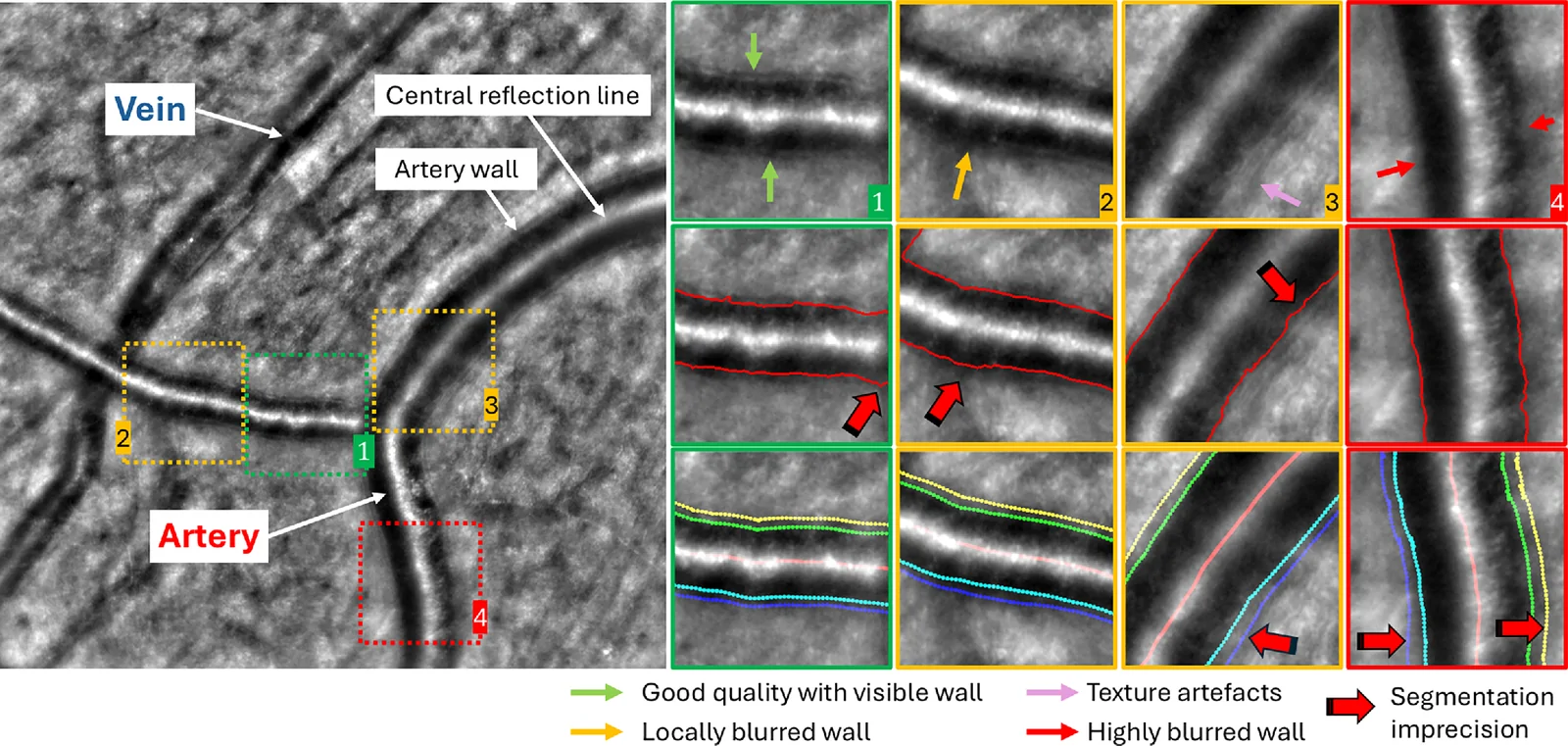
Example of a 1500 × 1500 AOO image with automatic segmentation results obtained with AOV.^27^ The four patches (first raw) highlight the varying quality levels within the image. The second raw shows the segmentation output by the DeepLabV3+ CNN and the third raw shows the final segmentation after the application of the active contour models. Segmentation accuracy is related to the local quality of the image, with segmentation imprecision often because of local blur or background artefacts.

The work by Sobczak et al.^6^ and Feng et al.^7^ provides useful clinical and physiological insights, demonstrating the usefulness of AOO imaging for in vivo detailed structural and functional analysis of the retina, and the influence of flicker on blood velocity and vessel dilation. However in these works, the delineation of vessels is done manually, which might be tedious and not always reproducible. Our method is therefore complementary to these works, and could help in further analysis, as done by these two groups, by relying on a robust automatic segmentation, less prone to user variability.

In the literature on automatic segmentation in this domain, most research works investigate deep-learning based methods to segment retinal vessels in conventional eye fundus images (retinography, confocal scanning laser ophthalmoscopy), generally with convolutional neural networks (CNN) based on the U-net model.^8^ Several improvements of the U-Net have been introduced to further refine segmentation accuracy. For instance, Residual U-Net incorporates residual connections to prevent gradient vanishing and improve feature propagation, making it particularly effective for detecting small and thin vessels.^9^^–^^11^ Similarly, Pyramid U-Net leverages a multiscale pyramid structure to extract and integrate features at different scales, allowing for better recognition of vessels of varying sizes.^12^^,^^13^ Another major enhancement involves integrating attention mechanisms^14^^,^^15^ into U-Net-based architectures, enabling the network to emphasize relevant regions and enhance the detection of small and low-contrast vessels while suppressing background noise. For example, Attention U-Net uses spatial or channel attention mechanisms that allow the model to focus on vessel structures while reducing interference from irrelevant features.^16^^–^^19^ More recently, transformers have emerged as a powerful component in vessel segmentation applications, owing to their ability to capture both local details and global dependencies within retinal images. Hybrid models combining transformer modules and U-Net-like architecture have also been proposed, demonstrating promising advancements in handling complex vascular structures.^20^^–^^22^ However, all these methods require adaptations to be applied to AOO images, which offer a much higher image resolution (about1 to 2 µm/pixel against 10 to 20 µm/pixel). In particular, the vascular walls must be delineated by two curves to accurately estimate the diameter of the vessel lumen and the thickness of the walls.

To this end, our team has worked on two complementary approaches to process AOO images. First, we investigated several CNN models to segment the vessel lumen^23^ and we found that the DeepLabV3+ model combined with the Xception backbone provides the best results among the tested architectures, included U-Net-based models.^24^ Second, we proposed to refine the obtained segmentation using dedicated parametric active contour models. The segmentation of the arterial wall relies on a geometric model in which four curves are defined with respect to the central reflection line. The energy functional to be minimized includes a classical gradient-image term, to attract the curves toward edges, but also prior structural knowledge, namely the approximate parallelism of the curves, in the form of a regularization term.^25^ The idea is to make the active contour more robust to textured background and local blur. In our preliminary work, every curve evolves with respect to the central reflection line, but independently of the others.^26^ More recently, we introduced couplings between the four curves to better model structural prior information and gain in robustness.^27^ The binary mask provided by our DeepLabV3+ CNN is used to initialize the active contour models. This combination of two complementary approaches, a “learning-based” one and a “structure-based” one, is particularly efficient. The first one allows to learn the great variability of AOO images in terms of topology, appearance and quality, while the second improves the segmentation accuracy, when correctly initialized, thanks to the explicit modeling of structural information. To the best of our knowledge, our two-step approach^27^ is the only algorithm that performs automatic artery segmentation in AOO images. Moreover, the use of parametric active contour models allows us to propose a semi-automatic mode where the expert can reinitialize an active contour to get a better segmentation result. This supervised mode has proved to achieve accurate and reproducible analysis of AOO images.^27^ An extra stage is also proposed to segment precisely the arterial bifurcations.^28^ Our framework for the analysis of AOO images, called AOV^27^ (adaptive optics vessel analysis), includes all these methods and enables medical experts to process large database for clinical studies. Figure 1 illustrates our approach through zoomed views of areas of various image quality.

In this article, we extend our methods for processing individual AOO images to series of AOO images, for the analysis of arterial segments (bifurcations are not considered). This is particularly interesting for the longitudinal monitoring of patients with certain systemic pathologies, for example the ones treated for vascular diseases (e.g., hypertension), for which high-resolution imaging of retinal vasculature can provide valuable morphological information.^4^ Instead of segmenting each image separately, we propose a joint segmentation process to exploit the cross-correlation between the images of the series, in order to be more robust to blur and background artefacts, and finally get more accurate and consistent segmentations. Given a series of *N* images spatially registered, our idea is to rely on the segmentation of the best-quality image to drive the segmentation of the *N* − 1 other images through inter-images couplings. Indeed, the robustness and the accuracy of the segmentation are highly dependent on image contrast along the arterial walls, even with our most advanced methods based on deep learning^24^ and dedicated active contour models,^27^ as illustrated in Figure 1. Therefore segmenting first the best-quality image maximizes the chance to get a good segmentation result, which will be then used to process images of lower-quality through an inter-image regularization term integrated in the energy functional of the active contour model. The structural couplings introduced in our active contour models^27^ have proven effective in driving the curve to the correct position in regions of very low local contrast (see for example patches 1 and 2 in Fig. 1); here, we extend this coupling principle to the temporal axis. This first requires to evaluate the AOO image quality over the region of interest in all images and to select the best-quality image that will provide reliable segmentation information.

In this article, we will illustrate our method on series of flicker images, for which the image quality issues are especially significant. Flicker series (Fig. 2) consist of a set of six images that are sequentially acquired before and during a series of flashes. The goal is to characterize variations in retinal vessel diameters due to the light stimulation in pathological and healthy cases,^3^^,^^5^^,^^29^ in order to evaluate the vasomotor activity. This application is especially challenging since images are acquired within a small time lapse according to a specific protocol, without the possibility of optimizing the quality of each image in the sequence. So we generally observe very heterogeneous quality levels within the series, even when focusing on a particular region of interest, as illustrated in Figure 2. At the same time, we want to assess subtle structural changes due to the light stimulation, which requires very accurate segmentations.

**Figure 2.**
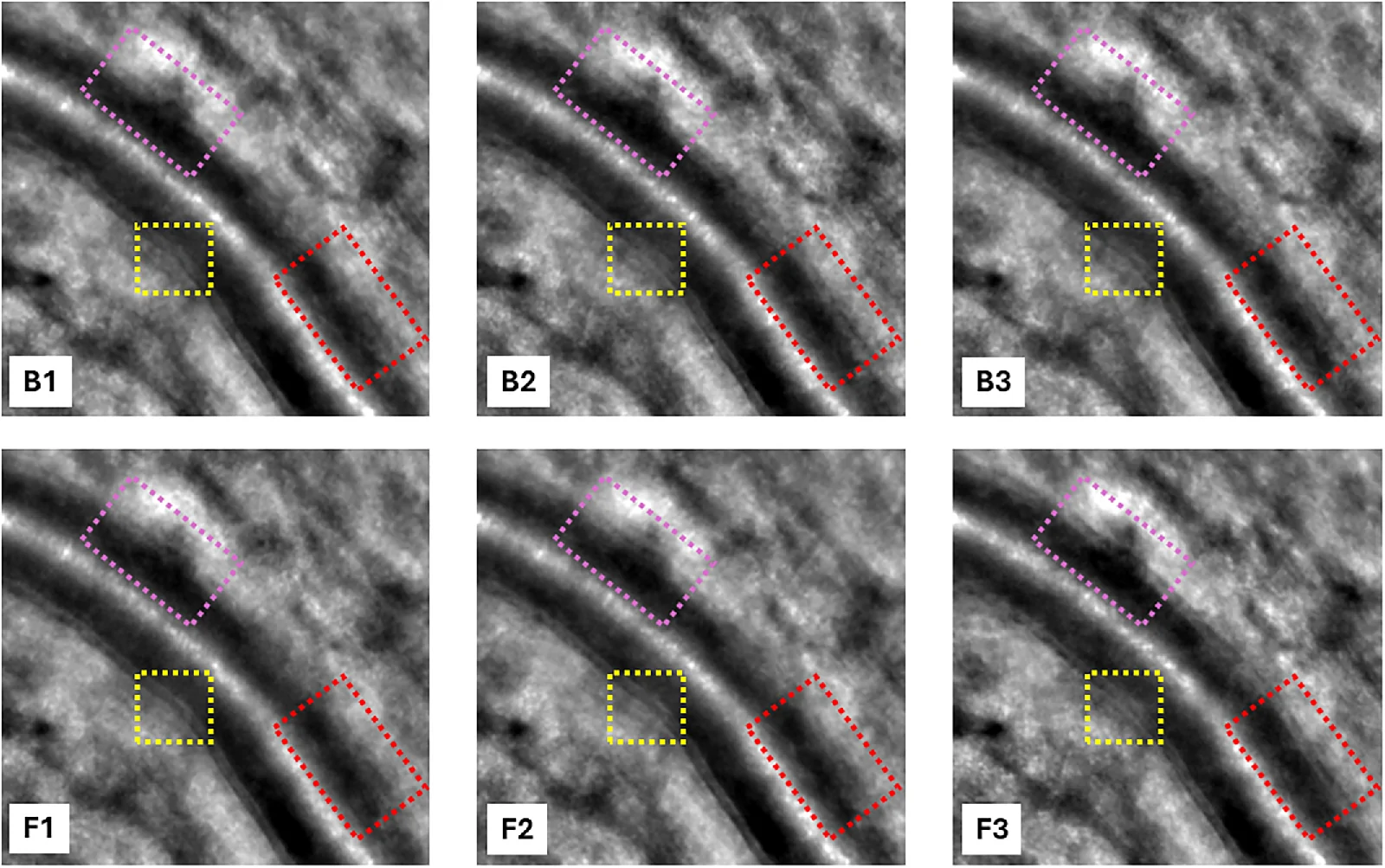
Example of six AOO images acquired before (B1, B2, B3, baseline) and during (F1, F2, F3) a flickering-light stimulus (flicker series). The colored boxes highlight differences in image quality around the artery walls. The accurate segmentation in the best contrast image (e.g., F3 in this case) can guide the segmentation in the images of lower quality.

To address all the above mentioned challenges, we propose a new image segmentation pipeline for the delineation of the retinal arterial walls in temporal series of registered AOO images, with an application focusing on flicker series to illustrate and evaluate our algorithm, since they are especially difficult to process automatically. In the pipeline, we assess the quality of every flicker image of the series by proposing a deep learning method and determine automatically the best quality image. Then we propose a two-step approach to segment the retinal arterial walls in the sequence, considering first the best quality image and then the five other images, with the temporal coupling mentioned above. This approach relies on the hypothesis that there is always a reasonably good-quality image in the sequence that can be automatically and accurately segmented with our methods.^24^^,^^27^ The image achieving the best quality score is referred to as the reference image and it is segmented first. Then additional couplings are proposed in the active contour model^27^ to segment automatically the five other images given the reference segmentation. These couplings ensure that the model evolves to a position that is consistent with the segmentation in the reference image, while adjusting itself according to the specificities of the processed image.

## Material and Methods

### AOO Flicker Image Dataset

#### AOO Technology

AOO is an image-improvement technology aimed at correcting high order optical aberrations due to ocular structures such as the cornea and lens. It is used in ophthalmology since the early 2000’s, and a commercial camera is available.^30^ Different imaging modalities can benefit from adaptive optics; here, we will only discuss AOO using flood-imaging, by opposition to scanning technologies such as scanning laser ophthalmoscopy. Flood AOO uses infrared light and captures a field of 4° × 4° (roughly 1.2 × 1.2 mm in emmetropic eyes) with a resolution around 2 µm which allows observing thin structures such as the retinal arterial wall^2^ (Fig. 3). The AOO images provided by the rtx1 camera (ImagineEyes^30^) are two-dimensional (2D) images of dimensions 1500  ×  1500 pixels with a pixel size of Δ = 0.8 µm. See Gocho's article^31^ for details regarding the image acquisition and preprocessing.

**Figure 3.**
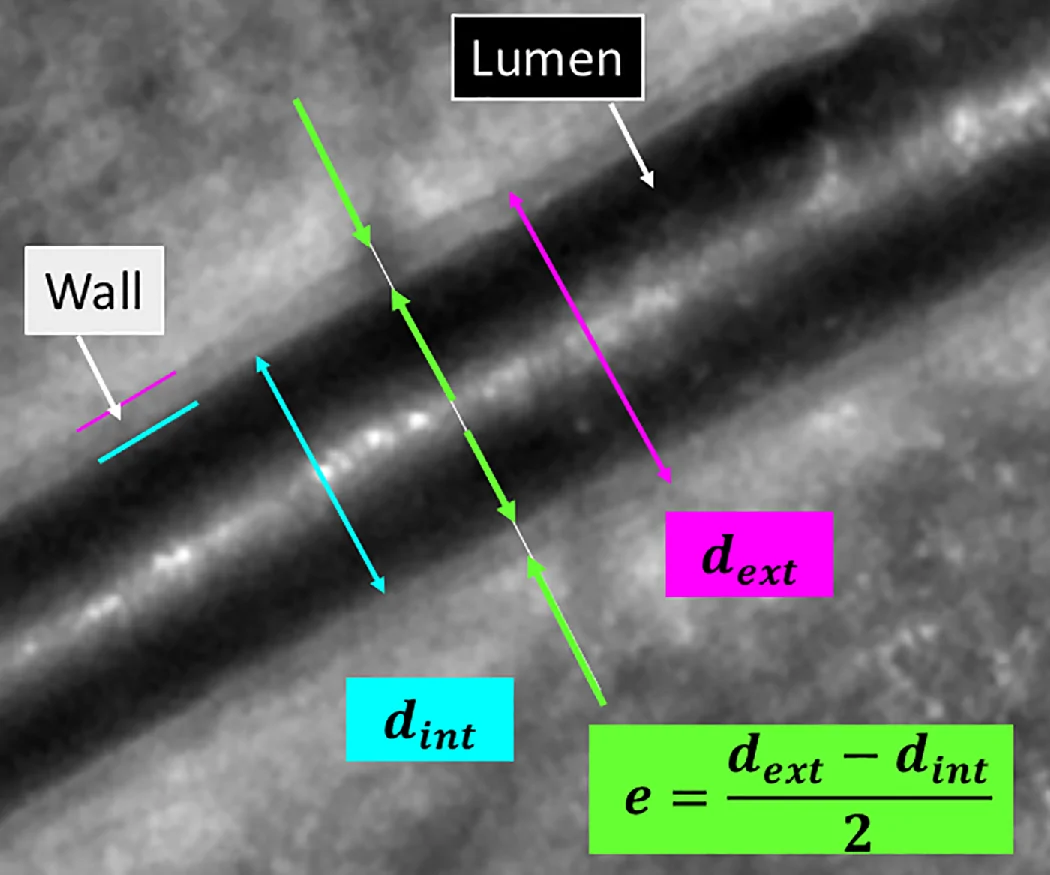
AOO image of a retinal artery and definitions (see also the text).

#### Acquisition protocol of AOO flicker images

A flicker device was implemented by the manufacturer in the rtx1 camera,^30^ delivering a full field (20°  × 28.8°) yellow flickering light (16 Hz,  4µ W/cm^2^) alternating with the fixation target during 22 seconds. As a consequence, the subject perceived flicker in both the background illumination and the fixation target. The continuous display of the live fundus image during stimulation ensured that fixation was maintained throughout the experiment. The protocol^5^ consists in capturing successive images of a vessel segment close to the macula, before and during stimulation, assuming that stimulated areas with abundant cones would result in more intense neurovascular coupling. Three successive baseline (unstimulated) images were acquired, followed by three images under flickering light stimulation during the last two seconds of the stimulus. Three cycles are performed to mitigate, by averaging, the effect of spontaneous fluctuations of vascular diameters called vasomotion. In a normal eye, this flicker stimulation elicits a 3% to 5% dilation of the artery. An illustration of a series of flicker images is given in Figure 2.

#### Measures Extracted From AOO Images

The lumen diameter *d_int_* (i.e., internal diameter) and the external diameter *d_ext_* (Fig. 3) are derived from the segmentation. The *d_int_* parameter is used to document flicker-related changes, as it is expected that the flicker induces a dilation of the lumen and consequently an increase of *d_int_*.

The wall-to-lumen ratio is defined as the ratio between the wall thickness *e* = (*d_ext_* − *d_int_*)/2 to the inner radius *d_int_*/2:

(1) $$\begin{array}{c} wlr=\frac{e}{d_{int}/2}=\frac{d_{ext}-d_{int}}{d_{int}} \end{array}$$

The *wlr* may indicate the degree of vascular tone, that is, constricted vessels show a higher thickness wall and a diminished lumen diameter. This parameter is robust to segmentation imprecision, especially for vessel calibers higher than 70 µm, as demonstrated mathematically and experimentally.^27^ It is of considerable interest in monitoring many pathologies, such as hypertension^4^^,^^32^^,^^33^ and diabetes,^34^ and their evolution under therapeutic treatment. Indeed, the *wlr* captures structural changes in the vessel wall relative to the lumen, providing a normalized measure of microvascular remodeling, whereas the lumen diameter alone can vary due to other factors. In systemic arterial hypertension, studies have consistently shown higher *wlr* in hypertensive subjects compared to normotensive controls^4^^,^^32^ and that the *wlr* decreases with blood pressure lowering through antihypertensive treatment.^33^ This confirms its clinical utility for longitudinal monitoring of hypertensive vascular damage.

The wall cross-sectional area (WCSA) measures the area of the artery wall, that is the difference between the artery section and the lumen section. So, it measures the volume of the vascular tissue at a given point. It is estimated by:

(2) $$\begin{array}{c} WCSA=\frac{\pi }{4}\left({d_{ext}}^{2}-{d_{int}}^{2}\right) \end{array}$$

In our flicker application, we study the evolution of three measures, the lumen diameter *d_int_*, the *wlr* and the *WCSA*. The *d_int_* parameter directly measures the vasodilation induced by light stimulation. Analyzing also *wlr* variations allows us to assess the quality of the segmentation and therefore the accuracy of the extracted parameters. Indeed, blur in the image results in the disappearance of low contrast features, such as the outer limit of vessels. Showing that the *wlr* slightly decreases, or at least does not increase during vasodilation, suggests a posteriori that the segmentation of the arterial wall is accurate and confirms the trend observed for *d_int_*. The third parameter, the WCSA, is expected to remain unchanged in flicker series, since acute vasomotricity does not cause changes in the vascular structure. Therefore, it is a second indicator of the reliability of the segmentation and measurements, especially since it is very sensitive to segmentation imprecision.

#### Database and Supervised Segmentation

The data base is made of 38 series of flicker images acquired from 26 healthy patients, recruited at the Quinze-Vingts hospital (Paris, France) within a study that has been approved by an ethics committee (Comité de Protection des Personnes of Ile-de-France; ClinicalTrial.gov, no. NCT01546181). A medical expert did a supervised segmentation of all series with our software tool AOV.^27^ Vessel segments, specifically accounting well-defined walls with good contrast in at least one image, were chosen for segmentation and diameter calculation. Images were acquired from either the right or the left eye. For 12 patients, two image series were collected on the same day, either from both eyes or from distinct, non-overlapping retinal regions of the same eye, depending on clinical needs.

First, the expert defined the central reflection line of the vessel segment to process by a few points (Fig. 4a). A standard active contour model^35^ is then applied on the top-hat image^36^ (Fig. 4b) to interpolate these points and obtain a line $V_{}^{\left(R\right)}$ that fits the central reflection for all images (Fig. 4c). The discretization step for $V_{}^{\left(R\right)}$ is equal to one pixel, so Δ = 0.8 µm. This line, referred to as reference line in what follows, will be the same for all six flicker images.

**Figure 4.**
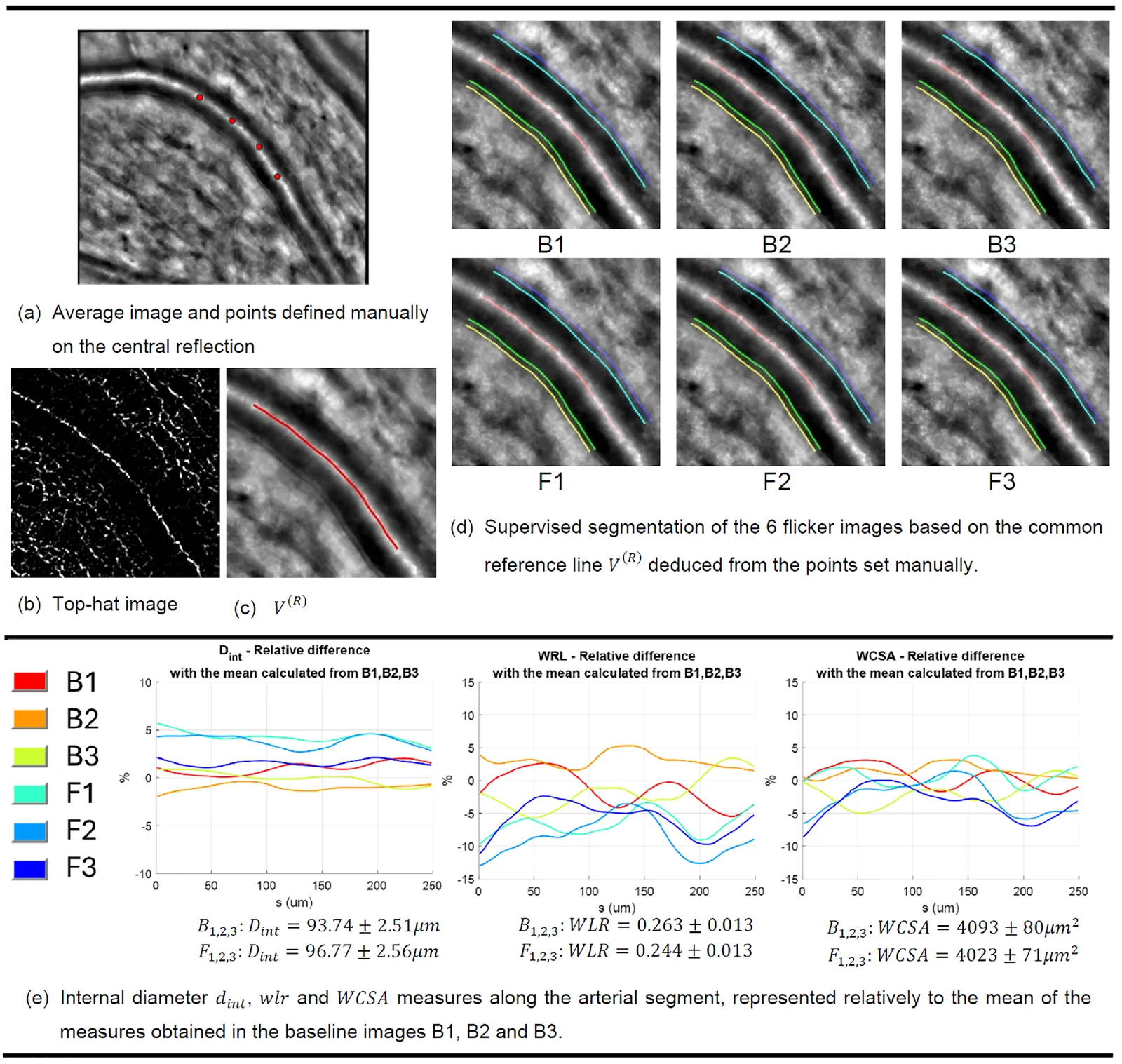
Example of a supervised segmentation of a series of flicker images with extracted morphometric parameters (same series as in Figure 2). (**a**–**c**): extraction of the central reflection line $V_{}^{\left(R\right)}$ from points manually defined; (**d**) Supervised segmentation of the arterial segment defined by $V_{}^{\left(R\right)}$ in the six images with AOV^27^; (**e**) Measures deduced from the segmented interfaces relatively to the mean obtained in the baseline images B. In the images captured under light stimulation (F1, F2, F3), we observe a significant increase in the lumen diameter, a decrease in the *wlr* while the WSCA does not show a significant trend.

Then each image is segmented under the expert supervision^27^ (Fig. 4d). The method is based on a dedicated parametric contour model, where all parametric curves are defined with respect to the reference line, considering the local distance of each point from it.^27^ In the supervised mode, the user can reinitialize manually the active contour model until satisfied with the final segmentation. This supervised segmentation algorithm achieves high accuracy and very good reproducibility of segmentations and biomarker estimations.^27^ For the flicker database, about 13% of the inner interfaces and 34% of the outer interfaces (23.5% in average) have been refined under the expert supervision. So, the results of this supervised processing of the flicker database will serve as ground truth in the experiments. Note that even though the six images have a common reference line, they have been then processed sequentially and separately. Figure 4 shows an example of a supervised segmentation of a flicker series with extracted measurements along the segment (Fig. 4e). It illustrates that small diameter changes can be captured within a flicker series.

### Image Processing Methods

The proposed image analysis pipeline for segmentation of the retinal arterial walls in a sequence of flicker AOO images is illustrated in Figure 5. Note that this pipeline also applies to any temporal series of spatially registered AOO images, only the number of images may change.

**Figure 5.**
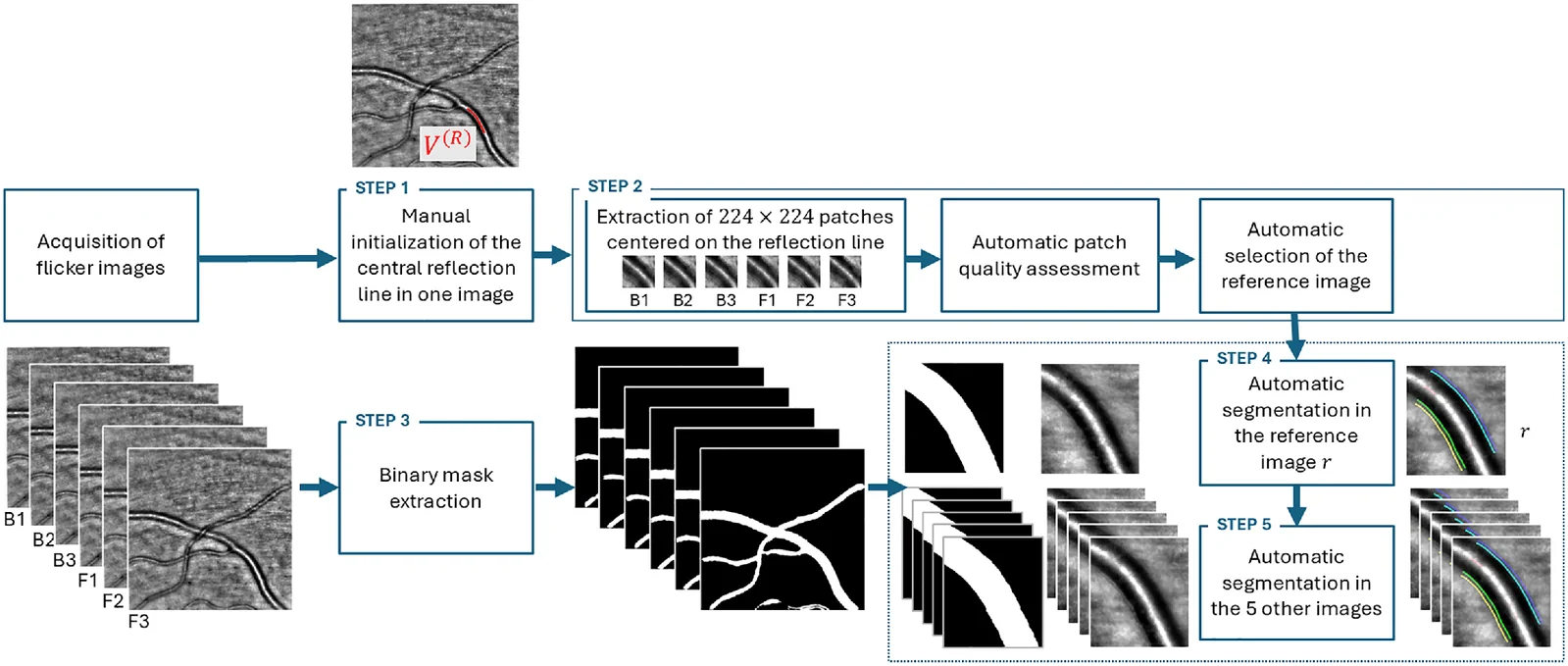
Segmentation of retinal arterial walls in a sequence of AOO Flicker images.

The first step is manual: the user defines the central reflection line of the artery branch of interest by a few points, as previously described. In step 2, a region of interest is defined as the 224  ×  224 square centered on the branch and six sub-images are extracted by cropping the six flicker images. The quality of each cropped image is evaluated by a deep learning method and the image with the highest score is considered the best quality image. In step 3, the DeepLabV3+ model combined with the Xception backbone^24^ is applied to all images to extract the 6 binary masks, which will be used to initialize the active contour models in steps 4 and 5. In step 4, the best quality image is automatically segmented applying our dedicated active contour model.^27^ The initial contour points for the inner borders are given by the binary mask edges. In step 5, the remaining five images are also automatically segmented on the basis of this first segmentation: the same active contour model is applied, but with coupling terms to the best image segmentation, in order to guide the evolution of the active contours towards consistent segmentations. This helps to overcome the difficulties associated with the lack of contrast and the noise in lower-quality images. In step 6, the diameters and thicknesses of the arterial walls are estimated in the six images, based on the segmentation results, and the wall to lumen ratio (*wlr*) and *WCSA* are calculated. It should be noted that the patch size (224  ×  224) is enough for the flicker application and many other applications related to artery follow-up. However, processing larger zones would only require merging quality scores estimated from contiguous patches, knowing also that the segmentation task is independent of the image size.

#### Step 1: Initialization of the Region of Interest

This is the same process as that carried out by the expert to process the database in supervised mode and it is the only manual step in the workflow described in Figure 5. The user selects one of the six images and specifies the branch to be processed by a few points on the central reflection line (Fig. 4a). These points are linearly interpolated to form a first line approximately on the central reflection, denoted by $V_{}^{\left(R\right)}$. We average the six images and enhance the central reflection by computing the morphological top-hat^36^ of this averaged image. A classical active contour^35^ is applied to regularize and refine the position of $V_{}^{\left(R\right)}$ so that it precisely follows the central reflection line (Fig. 4c). This processing is very robust since the central reflection line is highly contrasted in the top-hat image (Fig. 4b). Note that this central reflection line will be common to all six images. A region of interest is defined as the 224  ×  224 square centered on the selected branch segment. Six sub-images corresponding to this region of interest are then extracted from the six flicker images, for the quality assessment of the AOO images at the studied branch.

#### Step 2: Quality Assessment of AOO Images

The quality of a medical image is a significant factor for precise diagnosis by experts or computer-aided systems. The decision about the quality of a medical image can be highly dependent on clinical purposes and the type of applications. For example, in screening applications,^37^^,^^38^ the whole image should be considered to ensure that clinical and diagnostic signs are not missed. On the other hand, to determine valid morphometric measurements, one may want to focus on parts of the image containing specific structures (e.g., vessel walls).^39^^,^^40^

In the past years, several methods have been proposed to assess image quality acquired from different medical imaging modalities that can be commonly categorized into three groups^41^^,^^42^: (1) image structure, (2) generic image quality indexes, and (3) combination of both image structure and generic image quality indexes. In the image structure-based methods, the image is segmented and anatomical structures of interest (e.g., blood vessels in eye fundus images) are determined, then specific characteristics of the structures are considered to evaluate the quality of the image.^43^^,^^44^ Methods based on generic image quality indices compute directly quantitative indices such as contrast, focus, illumination, color and texture, from which the quality is derived.^45^^,^^46^ In the combination methods, a mixture of both characteristics of structures and generic indexes is used to assess the quality of an image.^47^^,^^48^

Nowadays, CNNs are widely used for different image processing tasks including the quality assessment of medical images.^40^^,^^49^^–^^51^ Despite the promising results of CNNs for this task, choosing a suitable architecture and training the network is not always easy. Although image quality assessment was performed in other ophthalmic imaging domains such as eye fundus^41^^,^^52^^,^^53^ and optical coherence tomography (OCT),^54^ according to our knowledge, we could not find any reported study that addresses this problem in AOO images. In this section, we consider a deep convolutional neural network for quality AOO images.

##### Image Data Preparation

We used an independent database of AOO images acquired from 27 patients to design our system. Because the image quality along the vessel walls varies a lot in an adaptive optics image, we cropped each AOO image at different positions along artery vessels. We obtained a total number of 616 cropped images with different qualities. All cropped images have a size of 224  ×  224 pixels and intensities were normalized between 0 and 1. We also provided a score for each image that was ranked from worst quality (0) to best quality (1). We considered a 5 scoring scale by assigning the score “1” to an image when all of the four internal and external walls in the image were clearly visible and conversely an image got the “0” score when the vessel walls were not distinguishable. Intermediate scores 0.25, 0.50 and 0.75 were assigned to images according to the visibility of vessel walls. Some selected cropped AOO images with assigned scores from our dataset are shown in Figure 6. We split the set of 616 images into 506 images for training, 60 images for validation and 50 images for testing.

**Figure 6.**
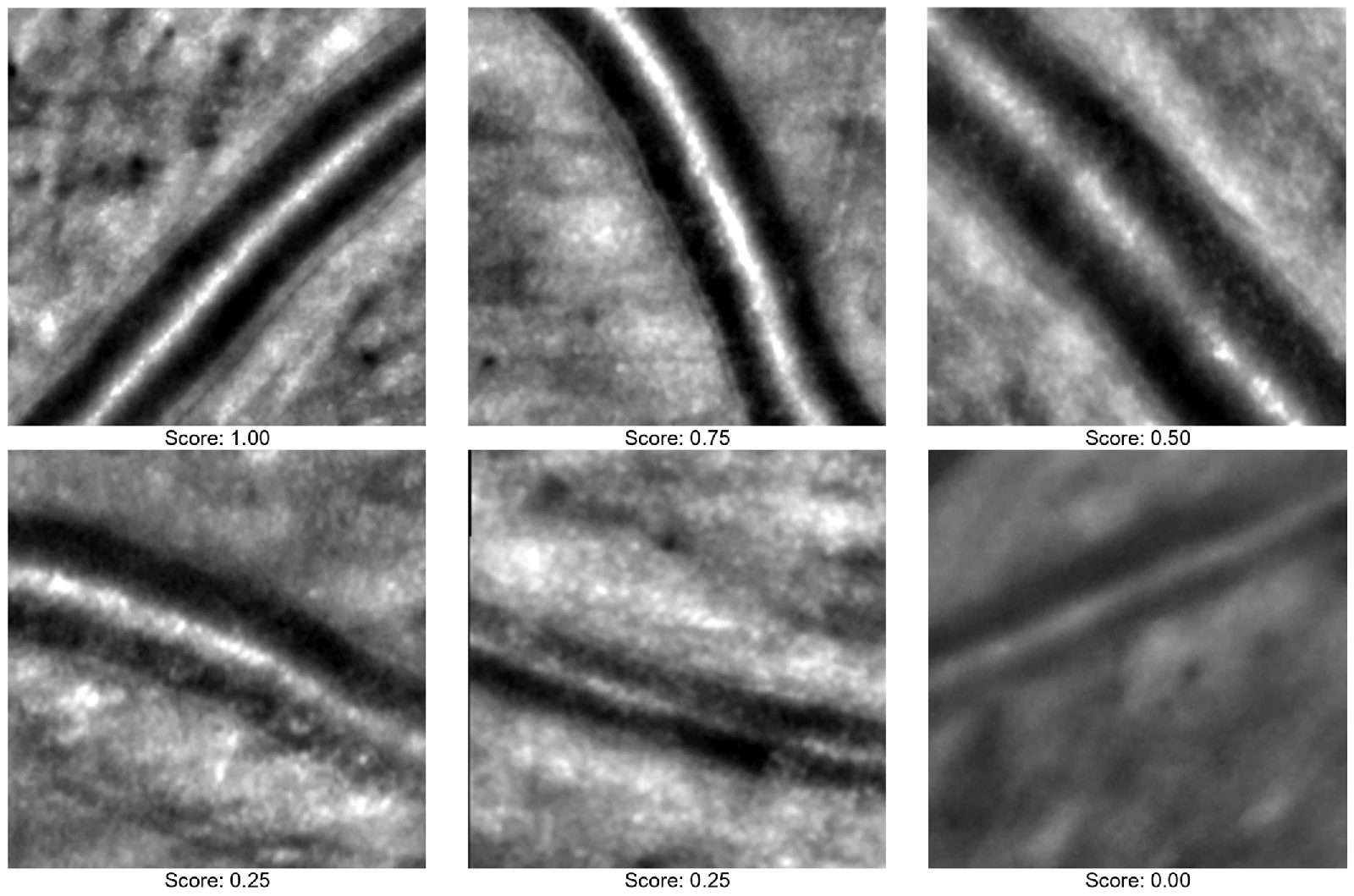
Cropped AOO images with assigned scores selected from our dataset used for quality assessment.

##### Model Architecture and Implementation

We propose using a deep CNN with transfer learning to assess the quality of AOO images. The model architecture deployed in this work is shown in Figure 7. We selected this model architecture inspired by previous work^49^^,^^53^^,^^52^ and after considering the performance of some different combinations of fully connected layers. In this model, at first, an AOO image is fed into a pretrained deep CNN model from which the last fully connected layers (classifier layers) have been removed. Then, the output of the pre-trained model is passed through two fully connected layers to predict the score of the image quality. We use the mean square error (MSE) as loss function since we consider the quality scoring as a regression problem.

**Figure 7.**
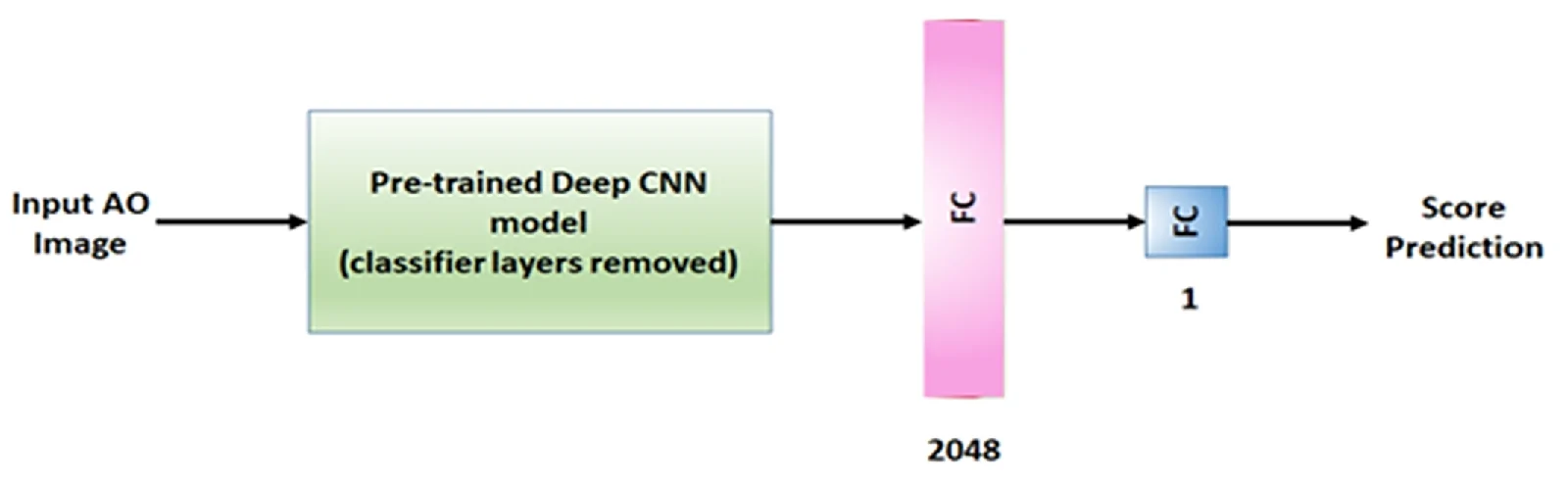
Model architecture used for quality assessment of adaptive optics in our work.

The proposed method was implemented in Python with the Keras package using the TensorFlow backend. We considered 8 popular pre-trained deep CNN models including: VGG16,^55^ VGG19,^55^ Inception v3,^56^ DenseNet121,^57^ Xception,^58^ InceptionResNetV2,^59^ ResNet50,^60^ and MobileNet.^61^ All of these models were pretrained on the ImageNet^62^ dataset. We employed these models as the baseline and fine-tuned them with our dataset in the proposed architecture to predict a quality score for the image. The two fully connected layers, with 2048 and 1 nodes, use a ReLU and a linear activation function, respectively.

To cope with the very limited size of the database in this research project, we artificially increased the number of images using data augmentation techniques: we applied rotation = 30°, zoom = 0.15, width shift = 0.2, height shift = 0.2, shear = 0.15, vertical and horizontal flipping. The networks were trained through stochastic gradient descent with a learning rate of 0.00001, with 100 epochs. By considering the experimental results (see the Section Results), we decided to select the Inception v3 as the best model and used it in the following steps of the proposed method. At inference, the scores of each of the flicker images are computed for each patient, and the image with the highest score is chosen as reference image, on which the segmentation is performed first.

#### Step 3: Extraction of the Binary Masks

The images are processed using the DeepLabV3+ architecture^63^ with an Xception backbone^58^ to extract the binary masks of the vessel lumen.^24^ This combination produces more consistent masks compared to those obtained with the basic U-Net architecture. The overall architecture is illustrated in Figure 8.

**Figure 8.**
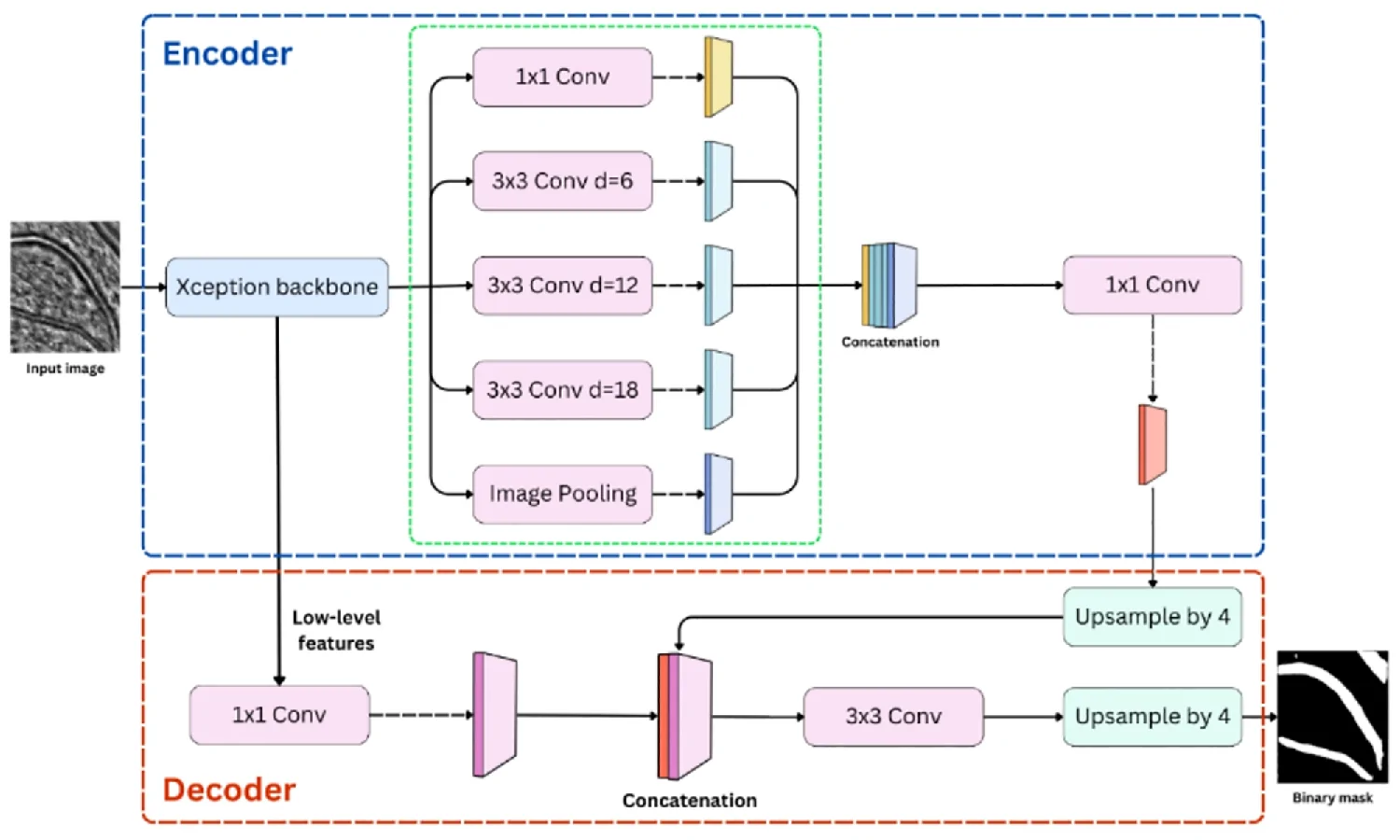
The DeepLabV3+ architecture with an Xception backbone. The Atrous Spatial Pyramid Pooling (ASPP) module is highlighted in green. d represents the dilation factor used in atrous convolutions.

The Xception backbone enables deeper feature extraction than U-Net standard convolutions, whereas the use of dilated convolutions in the Atrous Spatial Pyramid Pooling module allows for multi-scale feature processing, effectively capturing fine details. The segmentation is less sensitive to background artifacts and performs better in detecting complex structures and thin segments. Experiments showed an overall accuracy of 98.59 ± 0.04. However, the borders need to be refined and the external edge of the arterial wall is not yet segmented (Fig. 9).

**Figure 9.**
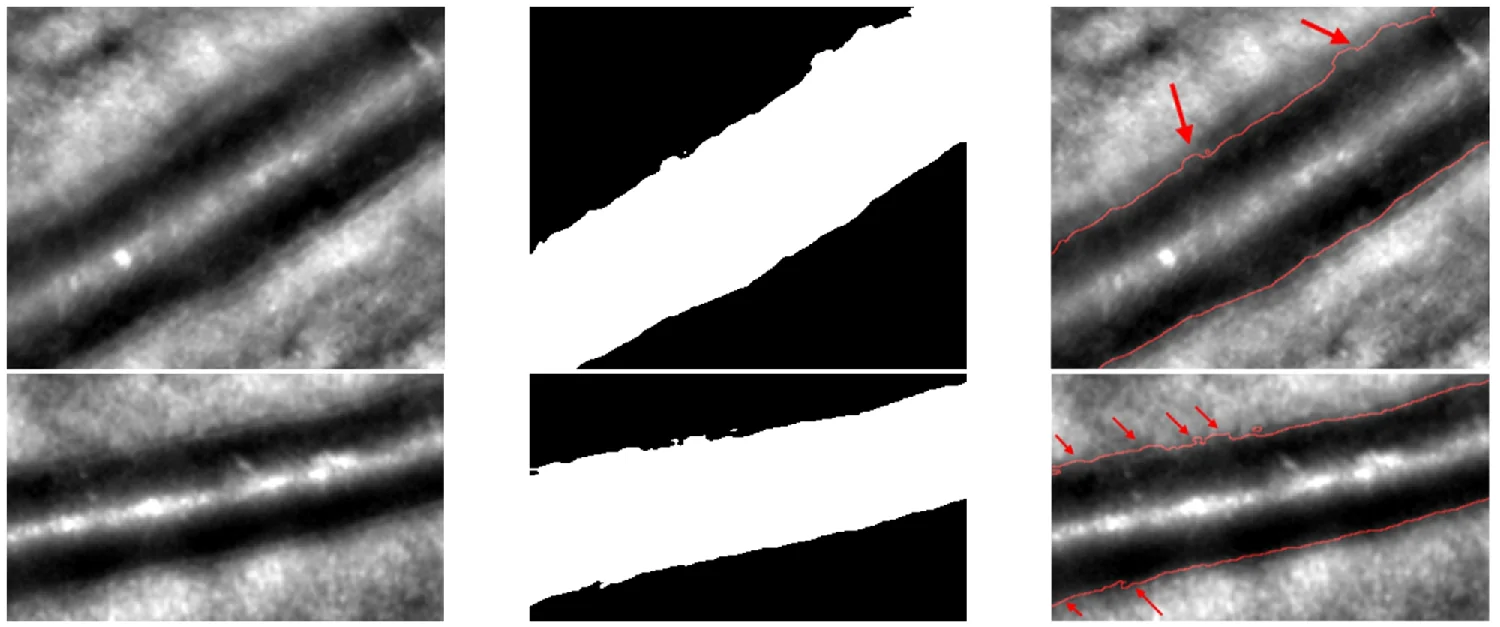
Two examples of the binary mask obtained with our DeepLabV3+ model.^24^ The images have been cropped on the region of interest. We observe inaccuracies in the delineation of the artery lumen despite the pretty good quality of the image in this area, justifying refinements thanks to the active contour models.

#### Steps 4 and 5: Segmentation of the Parietal Wall

We propose a dedicated active contour model to segment the arterial wall on either side of the central reflection line. In our model, each artery segment is represented by five lines: the central reflection line, two lines delineating the lumen (inner border of the arterial wall) and two lines on the outer border of the arterial wall.

As these five lines are approximately parallel, we propose to define the inner $V_{}^{\left(i_{1,2}\right)}\;$and outer contours $V_{}^{\left(e_{1,2}\right)}$ of the vessel wall by their distance to the central reflection line,^25^ denoted by $V_{}^{\left(R\right)}$ (Figure 10):

(3) $$\begin{array}{c} \begin{array}{c} \left\{{}_{V^{\left(i_{1}\right)}\;=\;V^{\left(R\right)}\;+\;b^{\left(i_{1}\right)}N^{\left(R\right)}}^{V^{\left(e_{1}\right)}\;=\;V^{\left(R\right)}\;+\;b^{\left(e_{1}\right)}N^{\left(R\right)}}\right.\;\left\{{}_{V^{\left(i_{2}\right)}\;=\;V^{\left(R\right)}\;+\;b^{\left(i_{2}\right)}N^{\left(R\right)}}^{V^{\left(e_{2}\right)}\;=\;V^{\left(R\right)}\;+\;b^{\left(e_{2}\right)}N^{\left(R\right)}}\right.\\ b^{\left(i_{1}\right)},b^{\left(e_{1}\right)}>0\;\;\;\;b^{\left(i_{2}\right)},b^{\left(e_{2}\right)}>0 \end{array} \end{array}$$

In these equations, $N_{}^{\left(R\right)}$ denote the unitary vector normal to $V_{}^{\left(R\right)}$, $b_{}^{\left(i_{1}\right)}\;$ and $b_{}^{\left(e_{1}\right)}\;$the distance from the inner and outer borders of the arterial wall to $V_{}^{\left(R\right)}$ for side 1, and $-b_{}^{\left(i_{2}\right)}\;$ and $-b_{}^{\left(e_{2}\right)}\;$ the distance from the inner and outer borders of the arterial wall to $V_{}^{\left(R\right)}$ for side 2.

**Figure 10.**
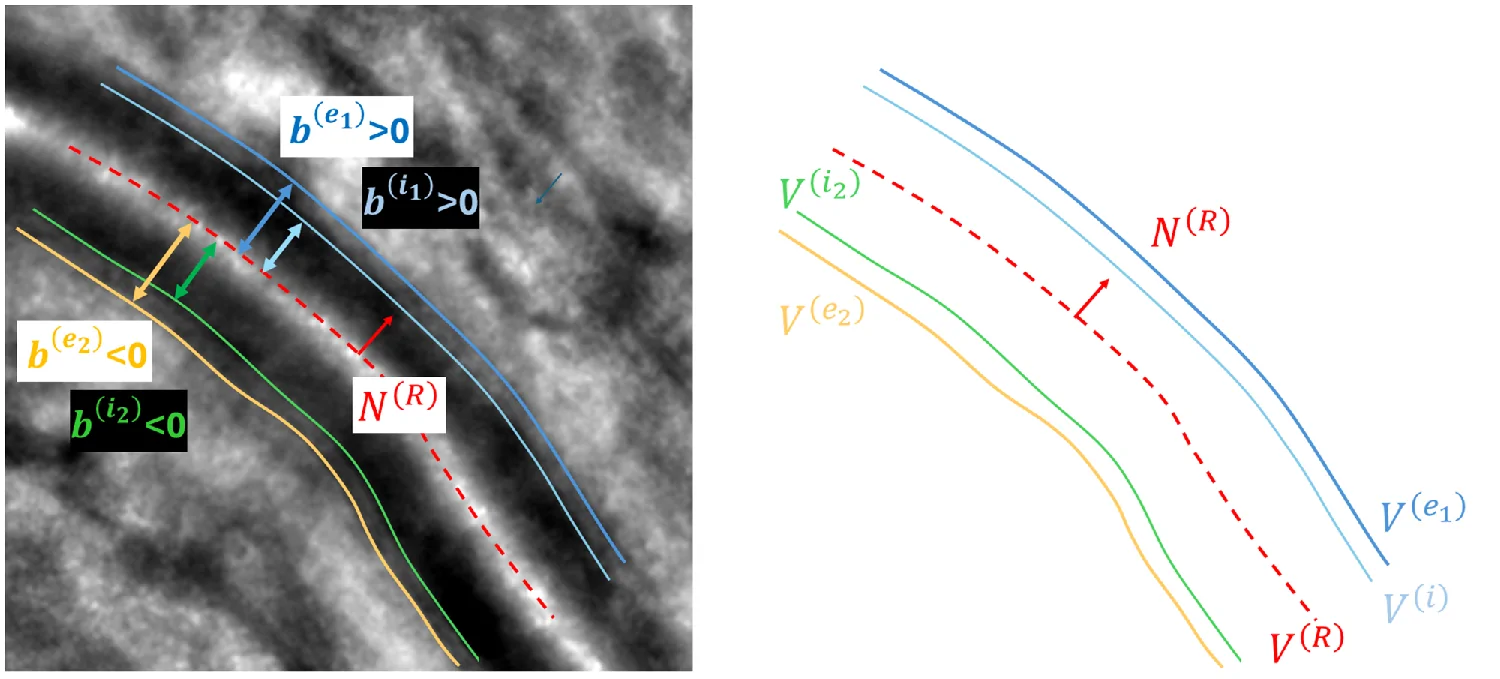
Geometric model of an arterial branch.

##### Generic Model

We propose a parametric active contour model that allows one curve to evolve with respect to the reference line under an approximate parallelism constraint and additional couplings with other lines.^27^ (Equation 3) introduces the general formulation of our model. In this equation, $V_{}^{\left(R\right)}$ is the reference line and all other lines,  *V*^(*i*,*k*, *l*)^ are defined by their local distance |*b*^(*i*,*k*, *l*)^| to it, *b*^(*i*,*k*, *l*)^ being positive (shift in the direction of the normal vector) or negative (shift in the opposite direction). The active contour is *V*^(*i*)^ whereas $V_{}^{\left(R\right)},\;V^{\left(k\right)}$ and *V*^(*l*)^ are known and fixed. The initial *V*^(*i*)^(*s*) is iteratively evolved to minimize the following energy functional:

(4) $$\begin{array}{ccc} & & \;E\left\{\;b^{\left(i\right)}|V_{}^{\left(R\right)},b^{\left(k\right)},b^{\left(l\right)}\right\}\\ & & =\int_{0}^{1}P\left(V_{}^{\left(R\right)}\left(s\right),b^{\left(i\right)}\left(s\right)\right)\\ & & \;+\frac{\varphi }{2}{b^{\left(i\right)}}^{{}^{'}2}\left(s\right)+\frac{\rho }{2}{\left(b^{\left(i\right)}\left(s\right)-b^{\left(k\right)}\left(s\right)\right)}^{2}\\ & & \;+\;\frac{\delta }{2}{\left({b^{\left(i\right)}}^{{}^{'}}\left(s\right)-\;{b^{\left(l\right)}}^{{}^{'}}\left(s\right)\right)}^{2}ds\; \end{array}$$

The first term, $P(\;V_{}^{\left(R\right)}\left(s\right),b^{\left(i\right)}\left(s\right))$, is the usual image potential at *V*^(*i*)^(*s*), defined as the gradient vector flow^64^ to attract the curve *V*^(*i*)^(*s*) to the high gradients in the image and so reach contours. The other terms are regularization terms that express structural constraints, namely:

– *V*^(*i*)^(*s*) and $V_{}^{\left(R\right)}\left(s\right)$ should be approximately parallel (which is expressed by the fact that the first derivative of the distance between them is minimized),
– *V*^(*i*)^(*s*) and *V*^(*k*)^(*s*) should be close to each other,
– *V*^(*i*)^(*s*) and *V*^(*l*)^(*s*) should be almost parallel.

The parameters φ,   ρ and δ weight the relative influence of all couplings. We rely on this model to segment the best quality image (i.e. the reference image) and the rest of the images.

##### Step 4: Segmentation of the Parietal Wall in the Best Quality Image

We apply our automatic segmentation method,^27^ with an initialization deduced from the binary mask. Let us denote by $V_{}^{\left(R\right)}$ the central reflection line common to all images, and by r∈[1,6] the index of the reference image. The inner borders $V_{r}^{\left(i_{1},i_{2}\right)}$ are initialized on the edges of the binary mask of image *r* and are evolved by minimizing:

(5) $$\begin{array}{ccc} & & E\left\{b_{r}^{\left(i_{1}\right)}|V_{}^{\left(R\right)},-b_{r}^{\left(i_{2}\right)},-b_{r}^{\left(i_{2}\right)}\right\}\\ & & \;+\;E\left\{b_{r}^{\left(i_{2}\right)}|V_{}^{\left(R\right)},-b_{r}^{\left(i_{1}\right)},-b_{r}^{\left(i_{1}\right)}\right\} \end{array}$$

This way, we encourage $V_{r}^{\left(i_{1}\right)}$ and $V_{r}^{\left(i_{2}\right)}$ to be approximately symmetrical with respect to $V_{}^{\left(R\right)}$ and favor similar shape for them. The binary mask provides a good initialization of the curves, especially when the image quality is good, whereas applying the active model effectively refines their position, leading to much better accuracy.^27^

Then we initialize the outer curves $V_{r}^{\left(e_{1},e_{2}\right)}$. This is done locally by shifting the inner curves on sliding and overlapping windows and selecting the shift that maximizes the mean local gradient module. Then we minimize

(6) $$\begin{array}{ccc} & & E\left\{\;b_{r}^{\left(e_{1}\right)}|V_{}^{\left(R\right)},-b_{r}^{\left(e_{2}\right)},b_{r}^{\left(i_{1}\right)}\right\}\\ & & \;+\;E\left\{\;b_{r}^{\left(e_{2}\right)}|V_{}^{\left(R\right)},-b_{r}^{\left(e_{1}\right)},b_{r}^{\left(i_{2}\right)}\right\} \end{array}$$

This time, we favor an approximate symmetry of the external curves and similar shape of the inner and outer borders.

A weighting strategy (φ,   ρ and δ) was optimized to achieve both robustness and accuracy.^27^ Selecting the image of best quality is crucial to achieve the most reliable and accurate segmentation possible, as this first segmentation will serve as a reference for processing the five remaining images.

##### Step 5. Segmentation of the Parietal Wall in the Five Remaining Images

Let us now consider the five other images, indexed by i∈[1,6]∖{r}. We rely on the segmentation found in image *r* by introducing inter-image couplings through the general formulation of our active contour model (Equation 4). The inner curves $V_{i}^{\left(i_{1}\right)}$ and $V_{i}^{\left(i_{2}\right)}$ are initialized from the binary mask of image *i* and then evolved by minimizing

(7) $$\begin{array}{ccc} & & E\left\{b_{i}^{\left(i_{1}\right)}|V_{}^{\left(R\right)},-b_{i}^{\left(i_{2}\right)},b_{r}^{\left(i_{1}\right)}\right\}\\ & & \;+\;E\left\{b_{r}^{\left(i_{2}\right)}|V_{}^{\left(R\right)},-b_{i}^{\left(i_{1}\right)},b_{r}^{\left(i_{2}\right)}\right\} \end{array}$$

This way, we again encourage the inner borders to be symmetrical with respect to the common central reflection line $V_{}^{\left(R\right)}$, but also the contours to be similar in shape with their counterparts in the reference image. This corresponds to a possible dilation of contraction of the vessel lumen between images *i* and *r*.

For the initialization of the outer curves, we consider that the arterial thickness does not significantly change because of the light stimulus. So, a good initialization is given by

(8) $$\begin{array}{ccc} & & b_{i}^{\left(e_{1}\right)\left[0\right]}=b_{i}^{\left(i_{1}\right)}+\left(b_{r}^{\left(e_{1}\right)}-b_{r}^{\left(i_{1}\right)}\right);\;\\ & & \;b_{i}^{\left(e_{2}\right)\left[0\right]}=b_{i}^{\left(i_{2}\right)}-\left(b_{r}^{\left(e_{2}\right)}-b_{r}^{\left(i_{2}\right)}\right) \end{array}$$

Then we minimize

(9) $$\begin{array}{ccc} & & E\left\{b_{i}^{\left(e_{1}\right)}|V_{}^{\left(R\right)},b_{i}^{\left(e_{1}\right)\left[0\right]},b_{i}^{\left(i_{1}\right)}\right\}\\ & & \;+\;E\left\{b_{i}^{\left(i_{2}\right)}|V_{}^{\left(R\right)},b_{i}^{\left(e_{2}\right)\left[0\right]},b_{i}^{\left(i_{2}\right)}\right\} \end{array}$$

So the intra-image coupling is again the similarity of shape of the inner and outer borders, whereas the model also encourages keeping the wall thickness found in the reference image.

### Estimation of Diameters, *wlr*, and WCSA

The segmentation of the inner and outer borders of the arterial wall enables us to calculate the wall-to-lumen ratio (Equation 1). This biomarker is evaluated along the artery branch. We apply a low-pass filter to the raw radius measurements to be more robust against local segmentation inaccuracies and to reveal only significant variations of these parameters. At each curvilinear abscissa *s_n_* = *n*Δ (Δ = 0.8 µm) on the central reflection line, we estimate the inner and outer diameters on a sliding window centered on *s_n_* and of size (2*K* + 1)Δ:

(10) $$\begin{array}{c} d_{int}\left(s_{n}\right)=\frac{1}{2K+1}\sum_{k=-K}^{K}b_{}^{\left(i_{1}\right)}\left(n+k\right)+b_{}^{\left(i_{2}\right)}\left(n+k\right)\; \end{array}$$

(11) $$\begin{array}{c} d_{ext}\left(s_{n}\right)=\frac{1}{2K+1}\sum_{k=-K}^{K}b_{}^{\left(e_{1}\right)}\left(n+k\right)+b_{}^{\left(e_{2}\right)}\left(n+k\right)\; \end{array}$$

(12) $$\begin{array}{c} wlr\left(s_{n}\;\right)=\frac{d_{ext}\left(s_{n}\right)-d_{int}\left(s_{n}\right)}{d_{int}\left(s_{n}\right)} \end{array}$$

(13) $$\begin{array}{c} WCSA\left(s_{n}\right)=\frac{\pi }{4}\left(d_{ext}{\left(s_{n}\right)}^{2}-d_{int}{\left(s_{n}\right)}^{2}\right) \end{array}$$

We set *K* = 30, so that the length of the window is close to 50 µm. Then the measurements are displayed as a function of the curvilinear abscissa *s_n_*, similarly to the graphs in Figure 4e.

## Results

### Evaluation of the Quality Assessment Method

Similarly to previous work,^63^^,^^65^^–^^67^ we calculated the Spearman rank-order correlation coefficient (SROCC)^68^ between the ranks of the obtained scores by our model (*r_M_*) and the ranks of the scores provided by the expert (*r_E_*), as follows:

(14) $$\begin{array}{c} SROCC\left(r_{M},r_{E}\right)=1-\frac{6\sum_{i=1}^{N}d_{i}}{N\left(N^{2}-1\right)} \end{array}$$

where *N* is the number of images in the test data and *d*_*i* _denotes the difference between the ranks of image *i* obtained by our model (*M*) and provided by the expert (*E*).

A comparison of the Spearman correlation coefficients computed on the test data for eight different pretrained models used as the backbone for the proposed method can be found in Table 1. As seen in this table, all pretrained models demonstrate a high correlation on agreement of relative image quality rank (*SROCC* ≥ 0.75) and the best result is obtained by the Inception v3 model (*SROCC* = 0.86). Therefore we choose this model to assess the quality of each of the 6 AOO images in the flicker sequence, and the image with the highest quality score is selected for the next steps.

**Table 1.** Calculated SROCC Using Different Pre-Trained Models for the Five-Scoring Scale

Pre-Trained Model	SROCC
VGG16	0.80
VGG19	0.75
Inception v3	**0.86**
DenseNet121	0.83
Xception	0.80
InceptionResNetV2	0.85
ResNet50	0.77
MobileNet	0.81

### Correlation Between Quality Scores and Segmentation Results

In this experiment, we evaluate the accuracy of the segmentation of each image of the flicker dataset, processed individually and automatically in AOV,^27^ and we evaluate the correlation with the quality score obtained with our DL approach. For that, we calculate the root mean square error between a segmented interface $\hat{V}$ and its counterpart *V* in the ground truth:

(15) $$\begin{array}{c} ɛ\left(V\right)=\sqrt{\frac{1}{L}\sum_{k=1}^{L}d{\left(\hat{V}\left(k\right),V\left(k\right)\right)}^{2}} \end{array}$$

where *d*() is the Euclidian distance and *L* the number of points on the curves. We consider the inner borders and the outer borders separately and we merge each time both sides in the root mean square error (RMSE) evaluation. Given a quality score $q_{n}\in \left[0,1\right]$, we consider an interval centered on *q_n_*, [*q_n_* − δ*q*,  *q_n_* + δ*q*], and we evaluate the probability that the RMSE ε(*V*) is less than 1 pixel, a criterion expressing the probability of a good segmentation. Figure 11a shows that this probability globally increases with the quality sore, especially for the outer border of the arterial wall, which suggests that the CNN effectively captures the contrast level of this thin structure. Figure 11b displays the box plot of the RMSE for the outer border. We can see that the median RMSE decreases as the quality score increases, with an increasingly lower dispersion. This experiment validates our idea to rely on the segmentation of the best quality image to do the segmentation of the other images of lower quality.

**Figure 11.**
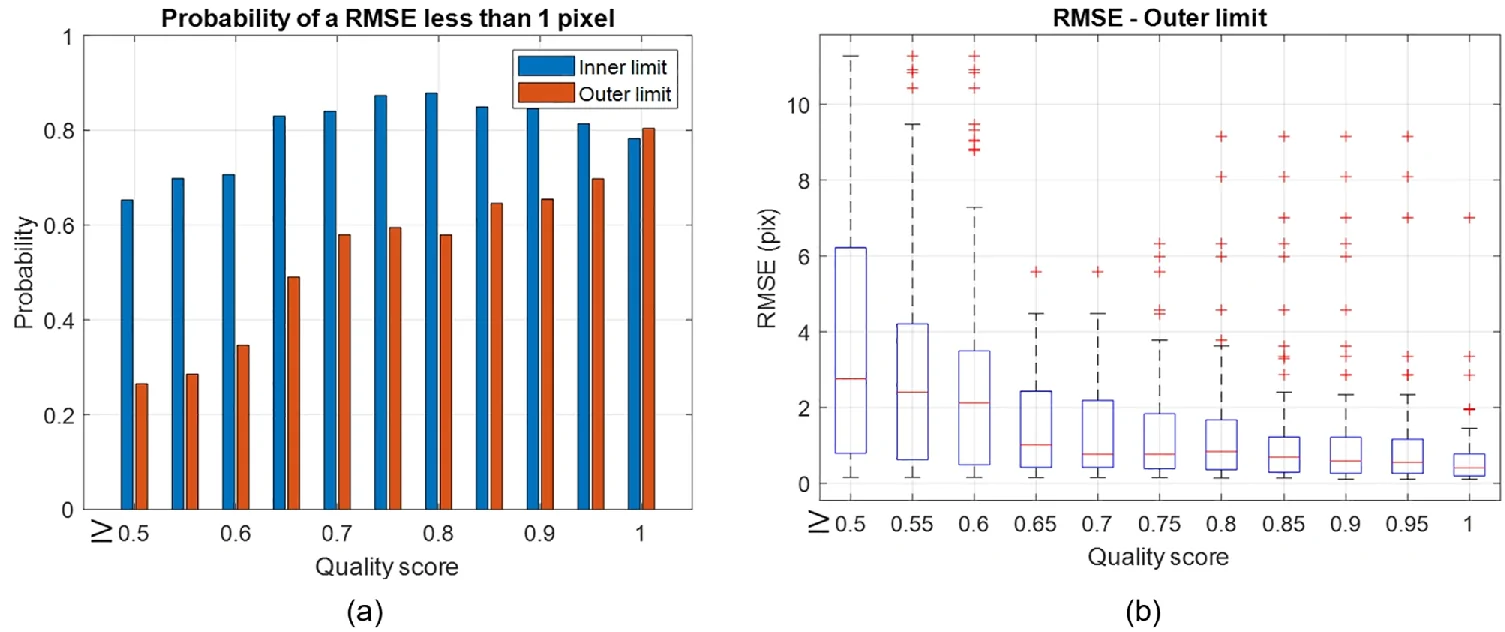
Correlation between the quality sore and the reliability and accuracy of the automatic segmentation result. (**a**) Probability that the RMSE is less than 1 pixel as a function of the Quality score. (**b**) Box plot of the RMSE as a function of the Quality score for the outer border of the arterial wall.

### Evaluation of Segmentation and Biomarker Estimation Results

We now propose to evaluate our joint time series image segmentation method in AOO on our flicker series dataset. We consider again the root mean square error between the reference segmentation made by the medical expert and the segmentation with the proposed method (Equation 15). We also evaluate the accuracy of the extracted features and biomarkers, namely the inner diameter *d_int_*, the outer diameter *d_ext_*, the wall thickness *e* and the wall to lumen ratio *wlr*. We did several experiments helping to understand the benefit of the quality assessment for the selection of the reference image and the impact of the inter-image couplings considering the segmentation in the reference image.

0. Baseline (Experiment 0): automatic segmentation of each image separately^27^ (step 4 applied to each image).

In all following experiments, we introduce the notion of reference image and the couplings to the segmentation in this image, with several levels of supervision.

1. Highly supervised mode (Experiment 1): the user defines the reference image, runs the automatic segmentation (steps 3, 4), correct it if necessary thanks to the supervised segmentation mode of AOV^27^ and runs the automatic segmentation of the five remaining images (steps 3, 5).
2. Supervised mode (Experiment 2): the user defines the reference image, runs the automatic segmentation of the reference image (steps 3, 4) and the automatic segmentation of the five remaining images (steps 3, 5).
3. Automatic mode with quality assessment (Experiment 3). The reference image is automatically selected as the one reaching the highest quality score (step 2) and the segmentation is performed automatically according to steps 3, 4, and 5.

To further evaluate the benefit of the quality assessment, we also propose an experiment where the reference image is randomly chosen.

4. Random selection of the reference image (Experiment 4), automatic segmentation of the reference image (steps 3, 4) and of the five remaining images (steps 3, 5).

Finally, the last experiment corresponds to a targeted usage mode, where the reference image with its segmentation is submitted to the user, who checks and eventually corrects this segmentation, and then launches the automatic segmentation of all five other images:

5. Experiment 5: Selection of the reference image by DL (step 2), automatic segmentation of this image (steps 3, 4), supervision of this segmentation, automatic segmentation of all five other images according to (steps 3, 5).

Let us denote by $y_{i}^{\left(n\right)}\left(k\right)$ the ground truth of a measure (e.g., lumen diameter *d_int_*) made in the *i*^th^ image of the series *n* at the index *k* on the branch, the value being estimated from the supervised segmentation of the medical expert. Let us denote by ${\hat{y}}_{i}^{\left(n\right)}\left(k\right)$ the same measure realized in one of the experiments. We first estimate the mean error of this measure along the branch:

(16) $$\begin{array}{c} ɛ\left(y_{i}^{\left(n\right)}\right)=\frac{1}{L^{\left(n\right)}}\sum_{k=1}^{L^{\left(n\right)}}\left({\hat{y}}_{i}^{\left(n\right)}\left(k\right)-y_{i}^{\left(n\right)}\left(k\right)\right) \end{array}$$

where *L*^(*n*)^ is the number of points on the vessel central reflection line.

Then, we calculate the statistics of the measurements errors on each series, by computing the mean error over the images of the series with its standard deviation:

(17) $$\begin{array}{c} \mu_{{ɛ}_{y}}^{\left(n\right)}=E\left\{ɛ\left(y_{i}^{\left(n\right)}\right)\right\}=\frac{1}{6}\sum_{i=1}^{6}ɛ\left(y_{i}^{\left(n\right)}\right) \end{array}$$

(18) $$\begin{array}{c} \sigma_{{ɛ}_{y}}^{\left(n\right)}=\sigma \left\{ɛ\left(y_{i}^{\left(n\right)}\right)\right\}=\sqrt{\frac{1}{6}\sum_{i=1}^{6}ɛ{\left(y_{i}^{\left(n\right)}\right)}^{2}-\mu {{}_{{ɛ}_{y}}^{\left(n\right)}}^{2}}\; \end{array}$$

A low $\mu_{{ɛ}_{y}}^{\left(n\right)}$ indicates that the measures ${\hat{y}}_{i}^{\left(n\right)}$ are without bias in the series *n*, while a low $\sigma_{{ɛ}_{y}}^{\left(n\right)}$ indicates a low dispersion around $\mu_{{ɛ}_{y}}^{\left(n\right)}$. Both values are important since we need accurate measurements in all six flicker images to detect small stimulus-related changes. Then we average these values along all series to get a global overview on the accuracy and robustness of the measures extracted in every series of the whole database.

(19) $$\begin{array}{c} \bar{\mu_{{ɛ}_{y}}^{\left(n\right)}}=\frac{1}{N}\sum_{n=1}^{N}\mu_{{ɛ}_{y}}^{\left(n\right)} \end{array}$$

(20) $$\begin{array}{c} \bar{\sigma_{{ɛ}_{y}}^{\left(n\right)}}=\frac{1}{N}\sum_{n=1}^{N}\sigma_{{ɛ}_{y}}^{\left(n\right)} \end{array}$$

$\bar{\mu_{{ɛ}_{y}}^{\left(n\right)}}$ is also the mean error of the measure *y* for all images of the database (i.e., the $ɛ(y_{i}^{\left(n\right)})$ averaged on all 6*N* images).

We also compute the standard deviation of the mean errors obtained on the series:

(21) $$\begin{array}{c} \sigma_{\mu_{{ɛ}_{y}}^{\left(n\right)}}=\sqrt{\frac{1}{N}\sum_{n=1}^{N}\mu {{}_{{ɛ}_{y}}^{\left(n\right)}}^{2}-{\bar{\mu_{{ɛ}_{y}}^{\left(n\right)}}}^{2}} \end{array}$$

A low $\sigma_{\mu_{{ɛ}_{y}}^{\left(n\right)}}$ indicates a good robustness at the series level.

In our quantitative evaluation, we calculate the root mean squared errors (Equation 15) for the inner ($V_{}^{i_{1,2}}$) and outer ($V_{}^{e_{1,2}}$) borders in each image and the statistics given by Equations 19–21 for the inner diameter *d_int_*, the outer diameter *d_ext_*, the wall thickness *e* and the *wlr* (Table 2).

**Table 2.** Quantitative Evaluation of the Segmentation and Biomarker Extraction in Six Different Experiments

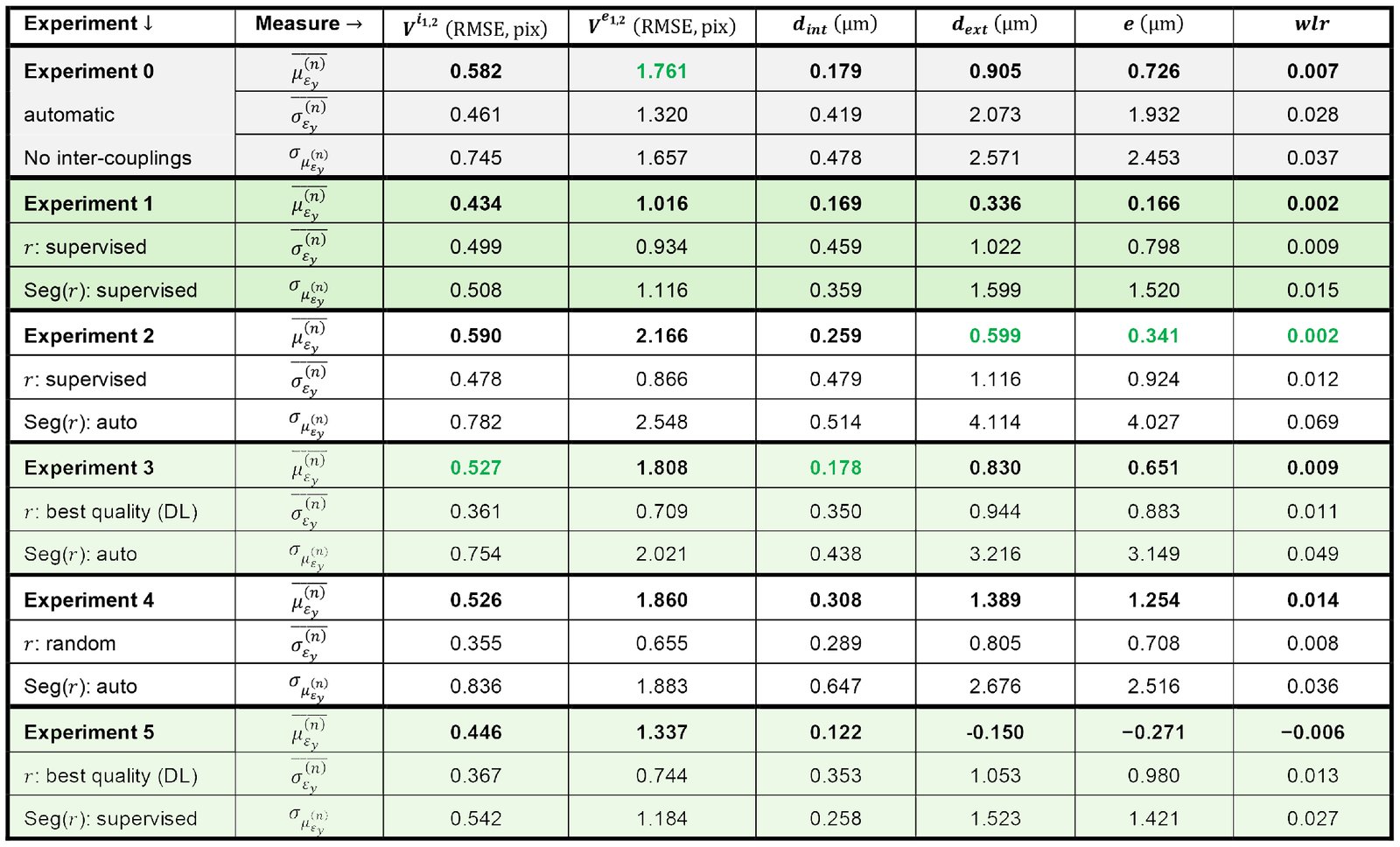

Best performance in bold.

In this quantitative evaluation, we chose to report measurement errors in pixels and micrometers, rather than expressing them relative to the lumen diameter. This allows errors to be directly interpreted across vessels of different calibers, given that the segmentation accuracy is limited to approximately half a pixel at each point, whereas relative errors would depend heavily on the vessel diameter.

In all experiments (Table 2), the segmentation of the inner borders is very accurate with mean errors and standard deviations less than one pixel. This means that the couplings to a reference image are not so important for the segmentation of the inner borders. As expected, the lumen diameter is also very accurately and robustly estimated. Experiment 3, with the reference image chosen by DL, achieves the best results among all experiments without supervision of the segmentation (Experiments 0, 2, 3, and 4), with a mean error of 0.18 ± 0.44 µm and a mean standard deviation in the series of 0.35 µm. This demonstrates that the proposed DL method selects a suitable image as reference image for the estimation of the inner diameter. However, inter-image coupling does not bring significant gain, as these performances are very similar to those of Experiment 0.

The segmentation of the outer interfaces is less accurate, with a mean error of 1.81 ± 2.02 pixels in Experiment 3, resulting in a mean error on the external diameter of 0.83 ± 3.22 µm. This reveals a small positive bias in the estimation of the external diameter (and so for the wall thickness, 0.65 ± 3.15 µm). Compared to experiment 0, the estimation of the outer diameter is in average better in Experiment 3 (lower mean error $\bar{\mu_{{ɛ}_{D_{ext}}}^{\left(n\right)}}$ and significant lower mean standard deviation $\bar{\sigma_{{ɛ}_{D_{ext}}}^{\left(n\right)}}$ (0.94 µm vs. 2.07 µm)) but with higher standard deviation through the series (3.22 µm vs. 2.57 µm), suggesting that the inter-couplings generally have a positive impact but may have a negative impact in a few series. Indeed, a bad segmentation in the reference image leads to bad segmentations in the five other images, although this is not the case without inter-couplings (Experiment 0). This shows the importance of a good choice for the reference image. An in-depth inspection through the series shows than inter-couplings with automatic selection of the reference image has a positive or neutral impact for 82% of the series (negative impact if the average loss is greater than 0.5 µm in the estimation of the outer diameter). The comparison with Experiment 4, where the reference image is chosen randomly among the six images, proves the importance of selecting an image of good quality to get a reliable reference segmentation that will drive positively the segmentation of the other five images: all measurement errors are in average significantly higher with a random selection, demonstrating the pertinence of the image quality assessment method and of the proposed workflow.

Figure 12 shows an example of series where the inter-image couplings (Experiment 3) have a global negative impact. However, we observe that they improve the segmentation on the vessel side where the arterial wall is visible, whereas the other side is too blurred to be segmented, even manually. Figures 13 and 14 present two other series with different quality levels. Despite the good overall quality of all images in the series in Figure 13, we observe that the proposed approach significantly improves the segmentation of both inner and outer borders, leading to much better measurements. In Figure 14, several images have low contrast on the arterial wall but the best image is indeed well selected and the inter-couplings bring a significant improvement.

**Figure 12.**
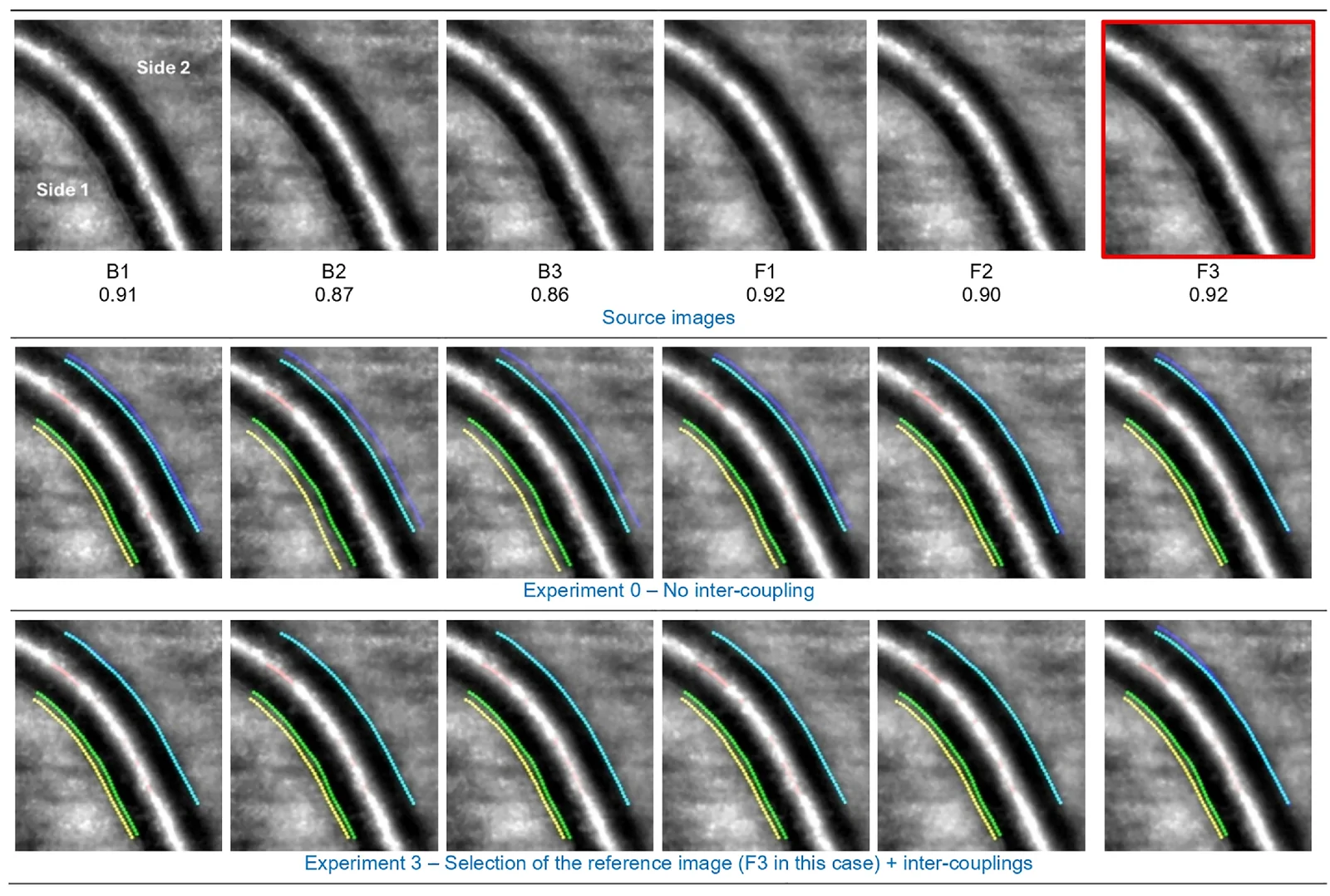
Example of a series where the inter-image coupling has a global negative impact, because of the wrong segmentation of the outer border on side 2 (*cyan/blue*) in the reference image (sixth image, F3). However, the image is too blurred on side 2 to be exploitable on this side. Conversely, inter-couplings bring a significant improvement in the segmentation on side 1 (*green/yellow*).

**Figure 13.**
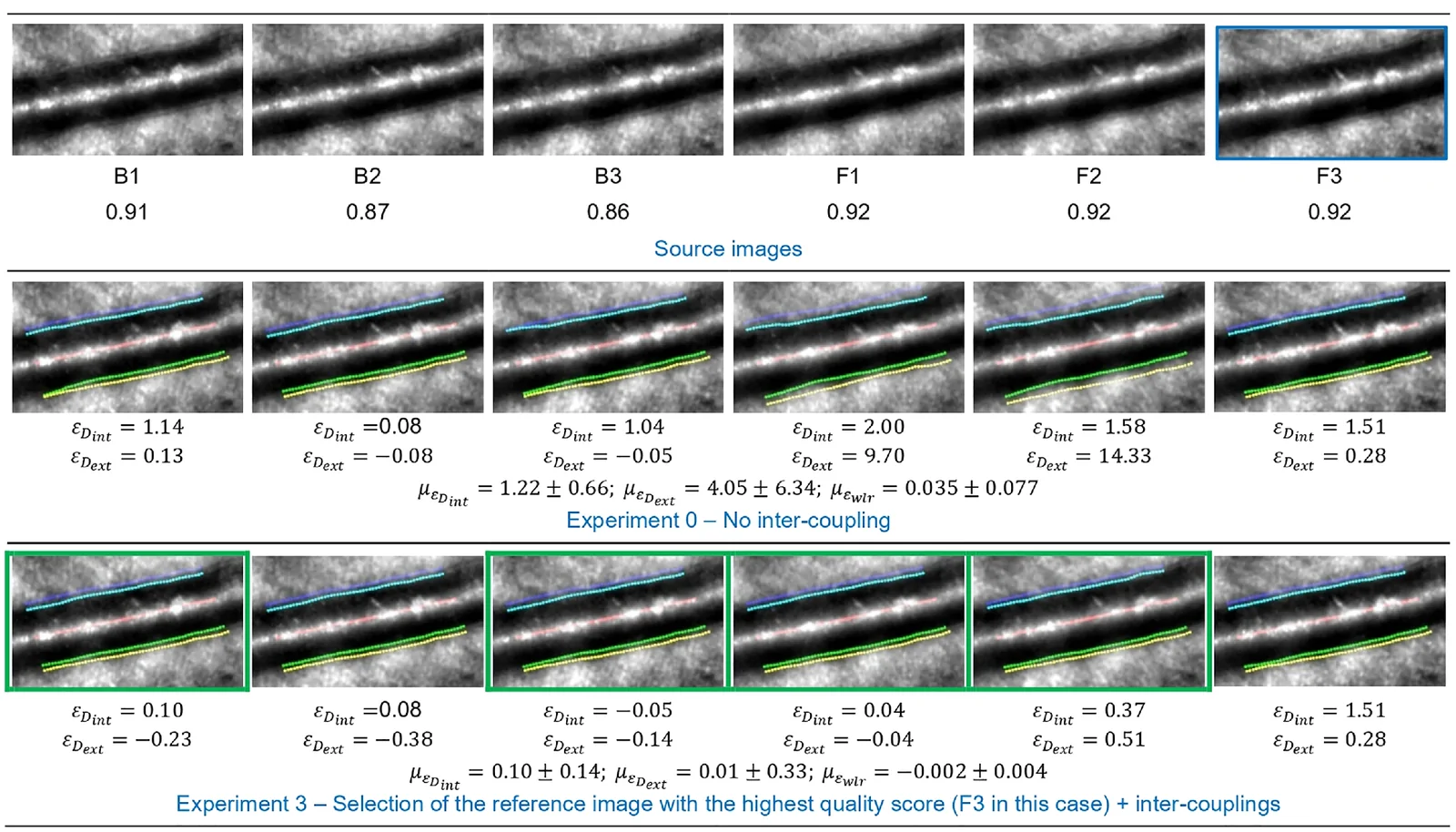
Example of a series with a good overall quality on the area of interest (see scores in the first row). Both inner and outer interfaces are much better segmented with the automatic selection of the best-quality image and the inter-image couplings (*green boxes* for a positive impact), leading to very accurate estimates of the diameters and *wlr*.

**Figure 14.**
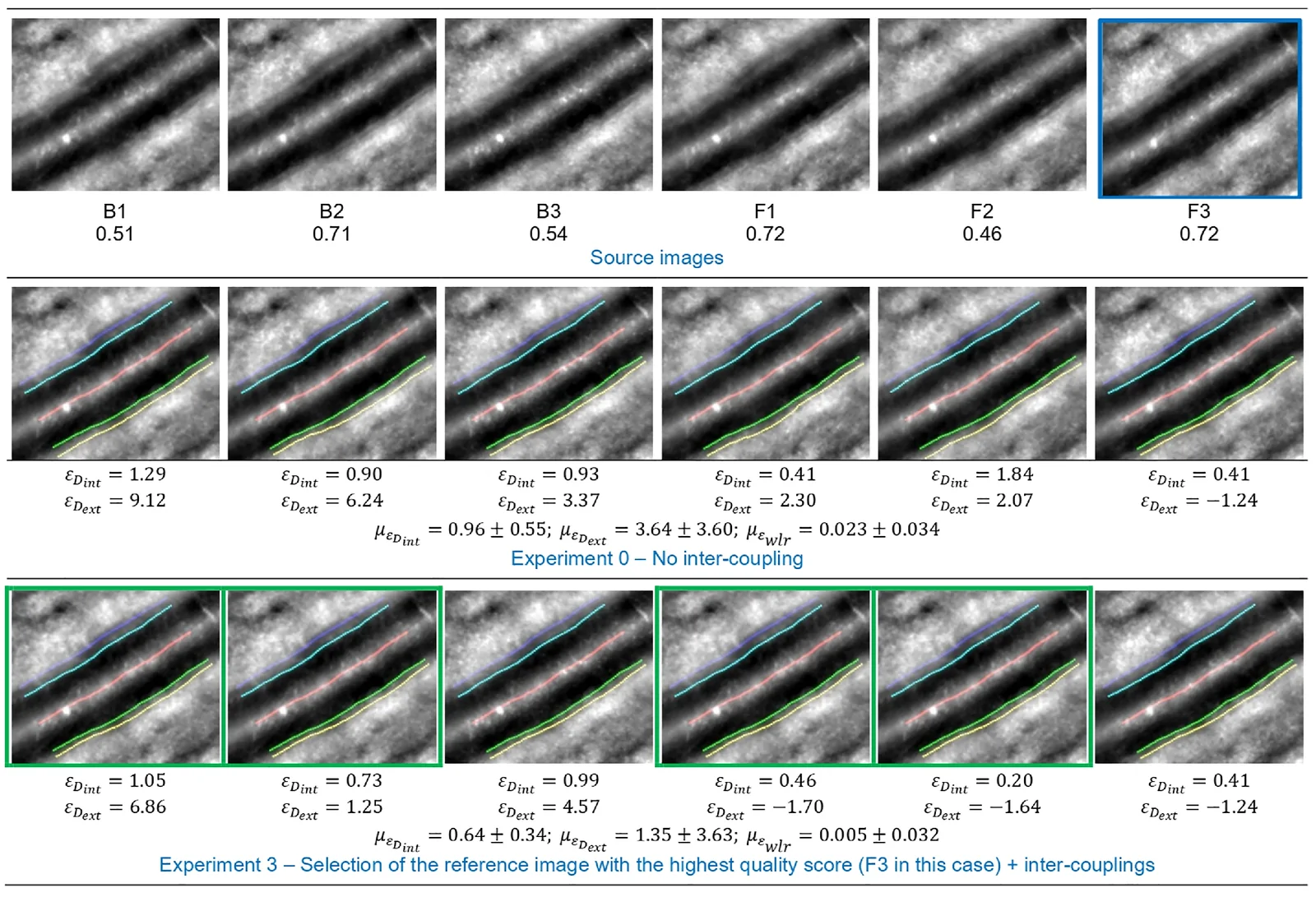
Example of a series with pretty good quality on the area of interest (see scores in the first row). The outer interfaces are much better segmented with the automatic selection of the best-quality image and the inter-image couplings, leading to very accurate estimates of the diameters and *wlr*.

Our analysis is corroborated by the results in Experiment 1, where the expert chooses the reference image and supervises its segmentation. The gain is very important: much better segmentation of the outer borders (1.02 ± 1.12 pix), much better estimation of the outer diameters (0.34 ± 1.60 µm), with a small mean standard deviation (1.02 µm) which proves that the segmentation within the series is very accurate once the segmentation of the reference image is good. So, the selection of a reference image and the supervision of its segmentation lead to very accurate and robust estimates of the inner and outer diameters, wall thickness, and *wlr*. This is a very important improvement since the medical expert has only to supervise one image out of six thanks to the inter-couplings and to perform a supervised segmentation in the few cases of failure: in Experiment 1, the user validated 96% of the interfaces segmented automatically in the reference images. Conversely, without inter-couplings, the expert must inspect all interfaces across all images. This approach was used to establish the ground truth, during which 23.5% of the interfaces required manual correction.

Finally, we did the last Experiment 5, where the reference image and its segmentation are submitted to the medical expert, who can correct the segmentation. It is therefore identical to Experiment 1, but the reference image is automatically chosen by DL (step 3). The segmentation performances are very similar to those of Experiment 1, so the selection of the reference image does not need to be checked by the user. It is worth noting that the *wlr* is very accurately estimated with a mean error of − 0.006 ± 0.027, knowing that the usual *wlr* values are generally in the range of 0.22 to 0.28.

Table 3 reports the MAE measurements for the experiments that implement the proposed joint segmentation method. These results demonstrate that the lumen diameter *d_int_* and *wlr* measurements are both accurate and reliable, supporting their use in clinical studies.

**Table 3.** MAE of the Measurements Derived From the Segmentations Performed With the Proposed Methods, in the Fully Automatic Mode (Experiment 3) and With the Supervision of the Reference Image Segmentation (Experiment 5)

Experiment ↓	Measure →	*d_int_* (µm)	*d_ext_* (µm)	*e* (µm)	*wlr*
Experiment 3	$\bar{\mu_{{ɛ}_{y}}^{\left(n\right)}}$	0.319	1.772	1.736	0.023
*r*: best quality (DL)	$\bar{\sigma_{{ɛ}_{y}}^{\left(n\right)}}$	0.288	0.795	0.708	0.009
Seg(*r*): auto	$\sigma_{\mu_{{ɛ}_{y}}^{\left(n\right)}}$	0.558	2.853	2.759	0.045
Experiment 5	$\bar{\mu_{{ɛ}_{y}}^{\left(n\right)}}$	0,267	1,141	1,125	0,015
*r*: best quality (DL)	$\bar{\sigma_{{ɛ}_{y}}^{\left(n\right)}}$	0,289	0,823	0,768	0,010
Seg(*r*): supervised	$\sigma_{\mu_{{ɛ}_{y}}^{\left(n\right)}}$	0,449	1,420	1,221	0,025

### Medical Application

Increasing precision and robustness and facilitating analysis will help AOO imaging of vessels to provide novel, clinically useful biomarkers to clinicians. Flicker-induced vasodilation has already been shown to be of interest in several clinical situations, some of which having no detected changes in the eye. Increasing precision of measures will ultimately not be only useful for diagnosis but also for monitoring diseases and therapy. Diseases affecting small cerebral vessel comprise a spectrum of pathologies that remains to be explored by AOO.

The flicker test was evaluated in a group of patients with a rare brain disease called CADASIL,^5^ using our segmentation framework AOV in the supervised mode.^27^ Among 98 patients evaluated, a significant decrease of the vasodilatory response to flickering light was found (1.6% vs. 3.6% in controls; *P* < 0.01). In this population, this test appears more sensitive than functional magnetic resonance imaging of the brain that provided inconclusive results on the same cohort of patients.

We now illustrate the automatic method proposed in this article for processing flicker series, taking as examples two specific cases, with one series from a control patient and another series from a patient with CADASIL. In both cases, the segment of interest is defined manually by the medical expert, and the segmentation is then performed automatically, according to the workflow described in Figure 5 (same as Experiment 3).

Figure 15 shows the segmentation of the control patient series. The local measurements derived from the segmentations (Equations 10–13) are averaged over each image and the results are then averaged on the three images B1-2-3 acquired before the light stimuli and over the three images acquired during (F1-2-3). A 4.0% increase in lumen diameter and a 7.7% decrease in *wlr* are observed. This corresponds to a significant vasomotor response, which is observable in the standard deviation image (Fig. 17, left). The curves of the lumen diameter and the *wlr* show a consistent and stable response along the arterial segment (Figure 18, first row). By contrast, the series acquired on the patient with CADASIL does not show a significant vasodilation (Figs. 16, 17, right). The average increase of the lumen diameter is neglectable (0.06%) and the curves are nested along the segment without any trend (Fig. 18, second row). The WCSA is quite stable in both cases, as expected, which indicates that the measurements are reliable. The results found in this experiment are consistent with the statistical study^5^ and illustrate the ability of the proposed methods to extract automatically accurate measurements from time series for medical applications.

**Figure 15.**
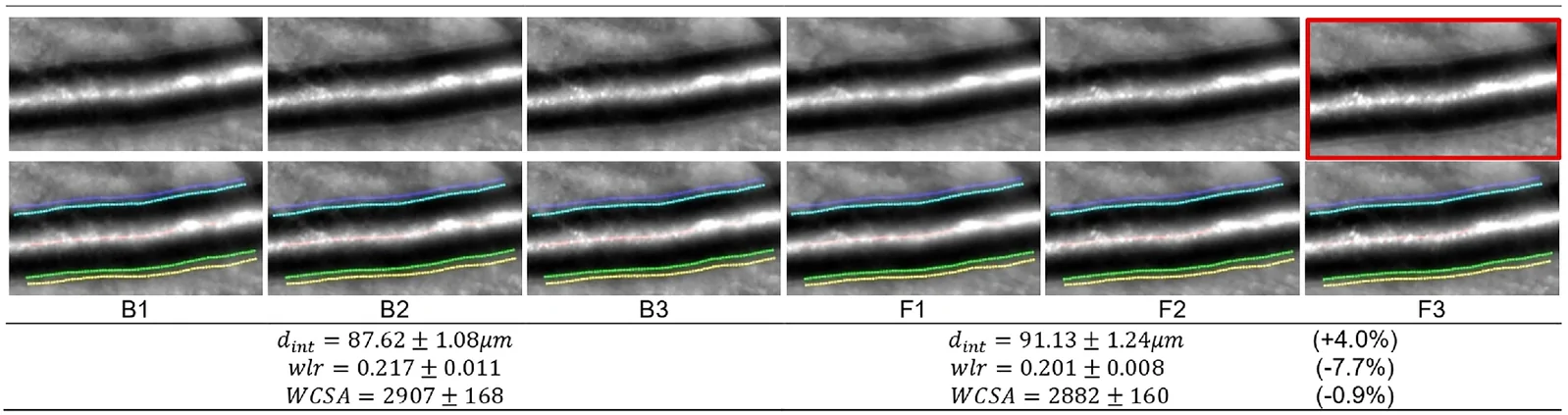
Example of a series acquired on a control patient.

**Figure 16.**
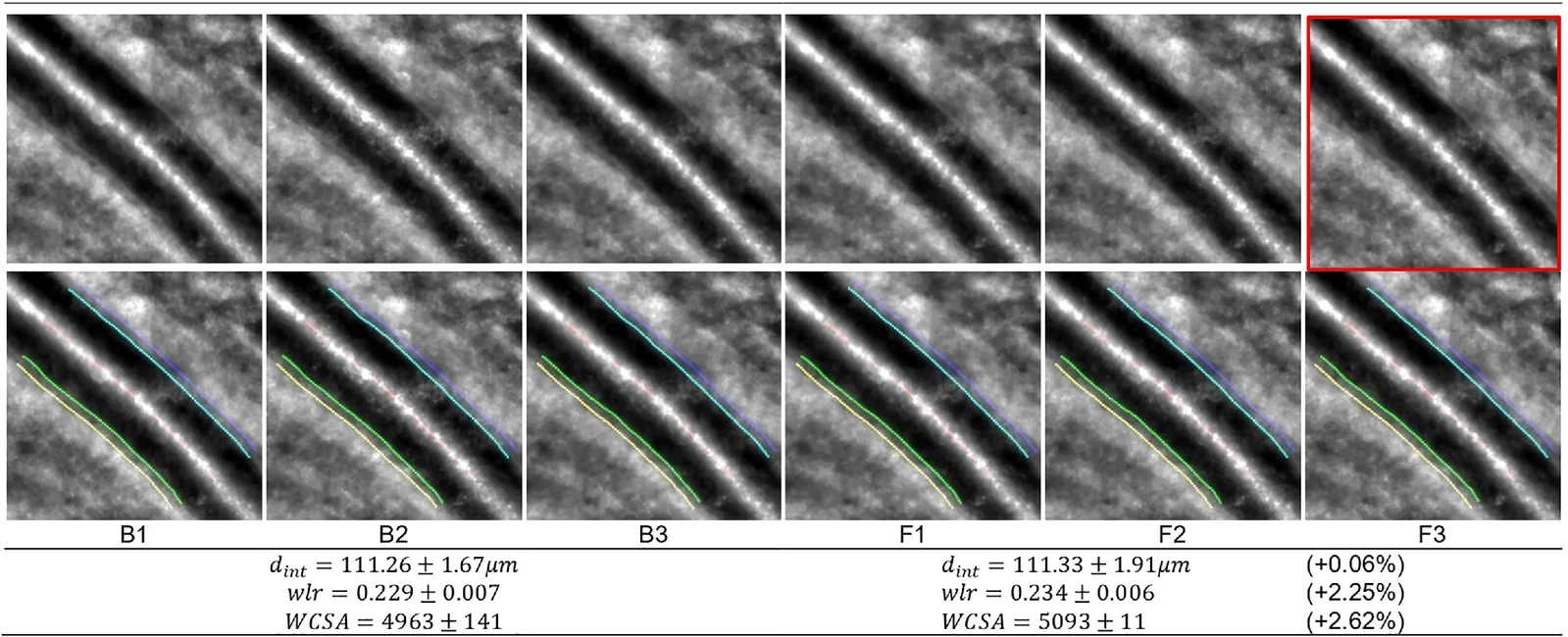
Example of a series acquired on a patient with CADASIL.

**Figure 17.**
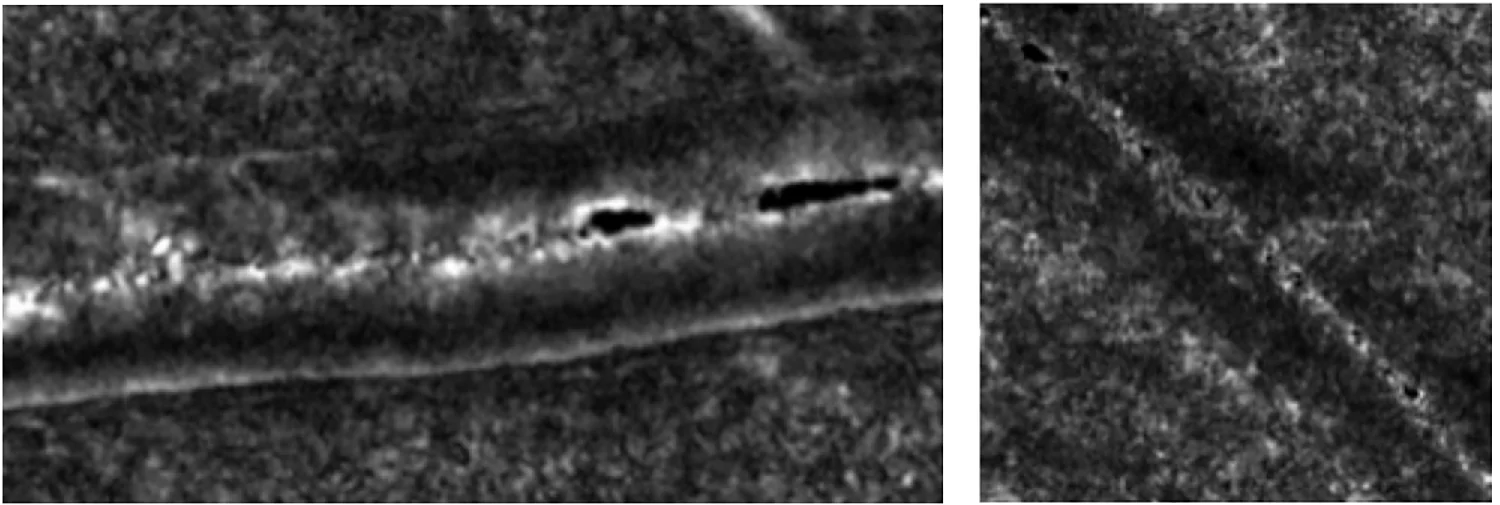
Standard-deviation image for the series of the control patient (*left*) and for the patient with CADASIL (*right*). The overbrightness along the vessel borders reveals a vasodilation in the control patient, whereas nothing is observable in the patient with CADASIL.

**Figure 18.**
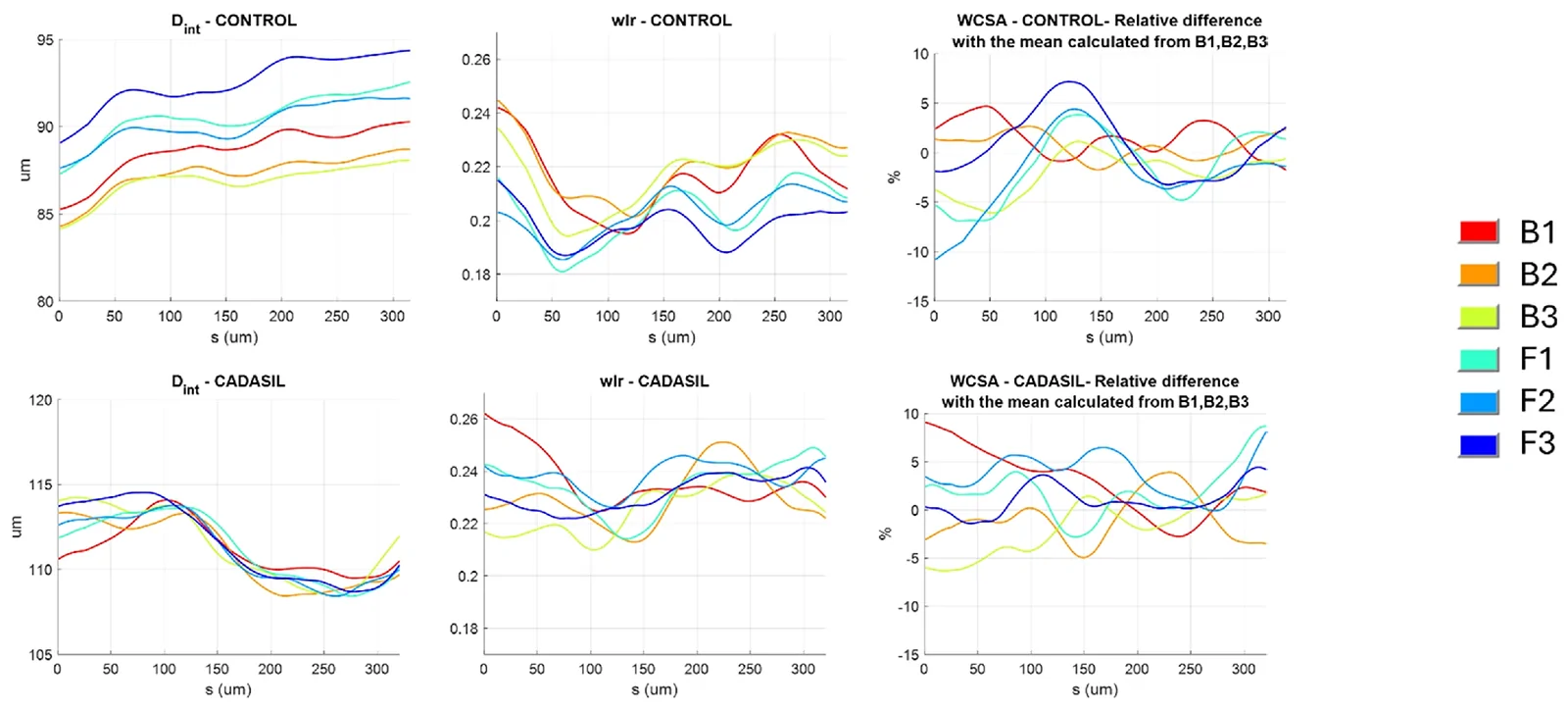
Lumen diameter *d_int_* and *wlr* measured along the arterial segment for a control patient and a patient with CADASIL. For the control subject, the curves show an increase of the lumen diameter and a decrease of the *wlr* along the segment, corresponding to a vasodilatory response to flickering light, while there is no significant or consistent trend for the patient with CADASIL. Relative variations of the WCSA compared to the mean over the baseline images are stable for both series (±10%) and do not show any particular trend, indicating that the measures are reliable.

## Discussion

We proposed an original segmentation method to jointly process AOO time series images and improve the reliability and accuracy of the extracted parameters while reducing user involvement. Our method is designed to process an arterial segment delimited by the user. Given a sequence of spatially registered images, we first determine the best quality image on the region of interest and segment it automatically. This segmentation then serves as a reference to segment all other images in the sequence, thanks to a dedicated active contour model integrating inter-images couplings.

We evaluated our approach on a database of flicker series, each series containing three baseline images and three images acquired under light stimulation. This application is especially challenging because the acquisition protocol does not allow to optimize the quality of each image through multiple acquisitions. A thorough qualitative and quantitative evaluation was performed, demonstrating the usefulness of the method: the selected artery branch is on average well segmented in the series, with a mean RMSE of 0.5 pixel (0.4 µm) and 1.8 pixels (1.44 µm) for the inner and outer borders of the arterial wall, respectively. This leads to a very robust and accurate estimate of the *wlr*. Even better accuracy is reached when the expert checks the segmentation of the reference image. This user involvement is very light and easily acceptable, as only a small rate of reference images needs to be corrected while this supervision guarantees very reliable automatic segmentation results for all the other images. In practice, initializing the reference line and verifying the reference segmentation takes less than a minute. All intermediate steps can be performed in batch mode. Correcting an interface, if necessary, takes about a minute; therefore, minimizing the frequency of this user intervention, thanks to the proposed method, is very valuable. Furthermore, we provided two examples of automatic processing of flicker images, a control case and a patient with CADASIL, and we demonstrated the ability of the proposed approach to observe and quantify vasoreactivity in a patient.

## Conclusions

Our algorithm can be used as a powerful and reliable computing tool for processing large time-series databases of AOO images. This study focusing on AOO images complements existing work on different types of imaging methods, such as fMRI, laser Doppler, OCT. It will help experts in their clinical research applications, to study the neurovascular coupling and also in the longitudinal patient follow-up. Indeed, evaluating long-term variations of vascular diameters is a promising perspective, one of the most interesting application being arterial hypertension. Future work will focus on automatic selection of the best region of interest, based on patch analysis over the complete binary mask and across the entire image series, to fully automate the process. Since the WCSA is immune to vasomotor processes, we plan to use it as a criterion for segmentation quality, which requires first studying its sensitivity to segmentation imprecision. Finally, we will extend our clinical studies to other small vessel diseases.
